# Supplementary material for: Mutations in GRK2 cause Jeune syndrome by impairing Hedgehog and canonical Wnt signaling
Source: EMBO Mol Med. 2020 Oct 14;12(11):e11739. doi: 10.15252/emmm.201911739 (PMC7645380; doi:10.15252/emmm.201911739)
Supplement: Supplementary file 1 — Appendix [file EMMM-12-e11739-s001.pdf]

Appendix

Appendix Table S1 **p. 2-6**

Appendix Table S1

Figure	Panel	Samples	Statistical test	N for statistics	p value (not significant)
3	C	Humerus, control vs. CMPD101	Mann-Whitney U test	20	8.0 x 10-9
		Ulna, control vs. CMPD101	Mann-Whitney U test	23	5.7 x 10-10
		Radius, control vs. CMPD101	Mann-Whitney U test	23	5.7 x 10-10
3	E	pH2.5, control vs. CMPD101 1uM	Mann-Whitney U test	5	0.0952
		pH2.5, control vs. CMPD101 2uM	Mann-Whitney U test	5(4)	0.0159
3	E	pH1.0, control vs. CMPD101 1uM	Mann-Whitney U test	5	0.42
		pH1.0, control vs. CMPD101 2uM	Mann-Whitney U test	5	0.0079
3	F	pH2.5, *Grk2*$^{+/+}$ vs. *Grk2*$^{-/-}$	Mann-Whitney U test	18	0.0052
		pH1.0, *Grk2*$^{+/+}$ vs. *Grk2*$^{-/-}$	Mann-Whitney U test	19	5.7 x 10-11
4	B	Control vs. Control + SAG	Mann-Whitney U test	4	0.021
		R05-365A vs. R05-365A + SAG	Mann-Whitney U test	4	0.89
4	C	GLI1; Control 100nM SAG vs. R05-365A 100nM SAG	Mann-Whitney U test	5	0.0317
		GLI1; Control 500nM SAG vs. R05-365A 500nM SAG	Mann-Whitney U test	5	0.0079
	C	PTCH1; Control 100nM SAG vs. R05-365A 100nM SAG	Mann-Whitney U test	5	0.0159
		PTCH1; Control 500nM SAG vs. R05-365A 500nM SAG	Mann-Whitney U test	5	0.0159
4	D	Control + SMO vs. R05-365A + SMO	Mann-Whitney U test	4	0.0027
		Control + SMO vs. Control + SMO + SAG	Mann-Whitney U test	4	0.35
		Control + SMO + SAG vs. R05-365A + SMO + SAG	Mann-Whitney U test	4	0.0022
		Control + GRK2 vs. R05-365A + GRK2	Mann-Whitney U test	4	0.0211
4	F	Control SAG 8hrs vs. R05-365A SAG 8 hrs	Welch's *t*-test	3	0.0029
		Control SAG 24hrs vs. R05-365A SAG 24 hrs	Welch's *t*-test	3	0.0218
4	G	Control vs. Control SAG 24 hrs	Mann-Whitney U test	56(56)	4.9 x 10-9
		R05-365A vs. R05-365A SAG 24 hrs	Mann-Whitney U test	55(54)	0.48
		Control SAG 24hrs vs. R05-365A SAG 24 hrs	Mann-Whitney U test	56(54)	6.6 x 10-7
5	B	Control vs. 500nM SAG	Mann-Whitney U test	59(59)	1.9 x 10-13
		CMPD101 20uM vs. CMPD101 20uM + SAG	Mann-Whitney U test	64(65)	1.2 x 10-12

		Control + SAG vs. CMPD101 20uM + SAG	Mann-Whitney U test	59(65)	0.0001
	B	Control vs. 500nM SAG	Mann-Whitney U test	62(62)	1.6 x 10-8
		Paroxetine 10uM vs. Paroxetine 10uM + SAG	Mann-Whitney U test	65(60)	0.00003
		Control + SAG vs. Paroxetine 10uM + SAG	Mann-Whitney U test	62(60)	0.0011
5	D	GLI3- FL/R; Control + SAG vs. CMPD101 20uM + SAG	Mann-Whitney U test	4	0.0286
		GLI3- FL/R; Control + SAG vs. Paroxetine 10uM + SAG	Mann-Whitney U test	4	0.0286
	D	GLI1; Control + SAG vs. CMPD101 20uM + SAG	Welch's *t*-test	3	0.0133
		GLI1; Control + SAG vs. Paroxetine 10uM + SAG	Welch's *t*-test	3	0.0188
5	E	shGRK2 vs. shGRK2 + Dox	Welch's *t*-test	3	0.0275
5	F	shSCR + SAG + Dox vs. shGRK2 + SAG + Dox	Mann-Whitney U test	65(71)	2.2 x 10-16
		shGRK2 + SAG vs. shGRK2 + SAG + Dox	Mann-Whitney U test	68(71)	2.2 x 10-16
6	A	Control + 100ng/mL Wnt3A vs. R05-365A+ 100ng/mL Wnt3A	Welch's *t*-test	3	0.0415
	C	LRP6/Actin; Control vs. R05-365A	Mann-Whitney U test	11	0.0077
	C	pLRP6^{T1572}/Actin; Control vs. Control + 30ng/mL Wnt3A	Mann-Whitney U test	5	0.0075
		pLRP6^{T1572}/Actin; Control vs. Control + 100ng/mL Wnt3A	Mann-Whitney U test	5	0.0075
		pLRP6^{T1572}/Actin; R05-365A vs. R05-365A + 30ng/mL Wnt3A	Mann-Whitney U test	5	0.056
		pLRP6^{T1572}/Actin; R05-365A vs. R0-365A + 100ng/mL Wnt3A	Mann-Whitney U test	5	0.0317
		pLRP6^{T1572}/Actin; Control + 100ng/mL Wnt3A vs. R0-365A + 100ng/mL Wnt3A	Mann-Whitney U test	5	0.095
	C	pLRP6^{S1490}/Actin; Control vs. Control + 30ng/mL Wnt3A	Mann-Whitney U test	5	0.005
		pLRP6^{S1490}/Actin; Control vs. Control + 100ng/mL Wnt3A	Mann-Whitney U test	5	0.0075
		pLRP6^{S1490}/Actin; R05-365A vs. R05-365A + 30ng/mL Wnt3A	Mann-Whitney U test	5	0.0317
		pLRP6^{S1490}/Actin; R05-365A vs. R0-365A + 100ng/mL Wnt3a	Mann-Whitney U test	5	0.0317
		pLRP6^{S1490}/Actin; Control + 100ng/mL Wnt3A vs. R0-365A + 100ng/mL Wnt3A	Mann-Whitney U test	5	0.0317
	C	DVL2/Actin; Control vs. R05-365A	Mann-Whitney U test	11	0.0006
		DVL2/Actin; Control vs. Control + 100ng/mL Wnt3A	Mann-Whitney U test	11	0.0006
		DVL2/Actin; Control + 100ng/mL Wnt3A vs. R05-365A + 100ng/mL Wnt3A	Mann-Whitney U test	11	0.0128
		DVL2/Actin; R05-365A vs. R05-365A + 100ng/mL Wnt3A	Mann-Whitney U test	11	0.44

	C	pDVL2/DVL2; Control vs. R05-365A	Mann-Whitney U test	6	0.0411
		pDVL2/DVL2; Control vs. Control + 100ng/mL Wnt3A	Mann-Whitney U test	6	0.0022
		pDVL2/DVL2; R05-365A vs. R05-365A + 100ng/mL Wnt3A	Mann-Whitney U test	6	0.0043
6	D	Control FZD4+ ARRB2 vs. R05-365A FZD4+ ARRB2	Mann-Whitney U test	70(62)	1.1 x 10-15
7	A	Grk2$^{+/+}$ vs. Grk2$^{-/-}$	Mann-Whitney U test	4	0.0019
	B	LRP6/Actin; Grk2$^{+/+}$ vs. Grk2$^{-/-}$	Welch's *t*-test	3	0.01
		LRP6/Actin; Grk2$^{+/+}$ + Wnt3A vs. Grk2$^{-/-}$ + Wnt3A	Welch's *t*-test	3	0.036
	B	pLRP6^{T1572}/Actin; Grk2$^{+/+}$ + Wnt3A vs. Grk2$^{-/-}$ + Wnt3A	Welch's *t*-test	3	0.148
	B	pLRP6^{S1490}/Actin; Grk2$^{+/+}$ + Wnt3A vs. Grk2$^{-/-}$ + Wnt3A	Welch's *t*-test	3	0.0128
7	C	LRP6/Actin; Control vs. CMPD101	Mann-Whitney U test	4	0.69
		LRP6/Actin; Control + Wnt3A vs. CMPD101 + Wnt3A	Mann-Whitney U test	4	0.28
	C	pLRP6^{T1572}/Actin; Control+ wnt3A vs. CMPD101 + Wnt3A	Mann-Whitney U test	4	0.021
	C	pLRP6^{S1490}/Actin; Control+ wnt3A vs. CMPD101 + Wnt3A	Mann-Whitney U test	4	0.021
7	D	LRP6/Actin; Control vs. CMPD101	Mann-Whitney U test	6	0.093
		LRP6/Actin; Control + Wnt3A vs. CMPD101 + Wnt3A	Mann-Whitney U test	6	0.93
	D	pLRP6^{T1572}/Actin; Control+ wnt3A vs. CMPD101 + Wnt3A	Mann-Whitney U test	6	0.0028
	D	pLRP6^{S1490}/Actin; Control+ wnt3A vs. CMPD101 + Wnt3A	Mann-Whitney U test	5	0.0075
7	E	Grk2$^{+/+}$ + FZD4 + ARRB2 vs. Grk2$^{-/-}$ + FZD4 + ARRB2	Mann-Whitney U test	70(70)	7.3 x 10-13
7	F	FZD4 + ARRB2 vs. FZD4 + ARRB2 + GRK2	Mann-Whitney U test	67(64)	4.4 x 10-16
EV1	B	Control vs. R05-365A	Mann-Whitney U test	155(154)	0.26
	C	24 hrs; Control vs. R05-365A	Welch's *t*-test	3	0.24
		48 hrs; Control vs. R05-365A	Welch's *t*-test	3	0.12
	F	R00-082 chondrocytes; Control vs. CMPD101	Mann-Whitney U test	78(75)	0.17
		R00-082 chondrocytes; Control vs. Paroxetine	Mann-Whitney U test	81(81)	0.08
	F	R92-284 chondrocytes; Control vs. CMPD101	Mann-Whitney U test	81(80)	0.58
		R92-284 chondrocytes; Control vs. Paroxetine	Mann-Whitney U test	83(87)	1.1 x 10-5

	G	R00-082 chondrocytes; Control vs. CMPD101	Welch's *t*-test	3	0.84
		R00-082 chondrocytes; Control vs. Paroxetine	Welch's *t*-test	3	0.08
	G	R92-284 chondrocytes; Control vs. CMPD101	Welch's *t*-test	3	0.44
		R92-284 chondrocytes; Control vs. Paroxetine	Welch's *t*-test	3	0.36
EV2	B	Control vs. Control + SAG	Mann-Whitney U test	61(59)	4.6 x 10-12
		CMPD101 20uM vs. CMPD101 20uM + SAG	Mann-Whitney U test	61(61)	5.6 x 10-16
		Control + SAG vs. CMPD101 20uM + SAG	Mann-Whitney U test	59(61)	0.1492
	B	Control vs. Control + SAG	Mann-Whitney U test	60(61)	1.8 x 10-8
		Paroxetine 10uM vs. Paroxetine 10uM + SAG	Mann-Whitney U test	62(62)	0.0011
		Control + SAG vs. Paroxetine 10uM + SAG	Mann-Whitney U test	61(62)	0.0002
	D	GLI3- FL/R; Control + SAG vs. CMPD101 20uM + SAG	Mann-Whitney U test	4	0.0286
		GLI3- FL/R; Control + SAG vs. Paroxetine 10uM + SAG	Mann-Whitney U test	4	0.0286
	D	GLI1; Control + SAG vs. CMPD101 20uM + SAG	Welch's *t*-test	3	0.0032
		GLI1; Control + SAG vs. Paroxetine 10uM + SAG	Welch's *t*-test	3	0.0075
EV3	A	$Grk2^{+/+}$ + SAG vs. $Grk2^{-/-}$ + SAG	Mann-Whitney U test	67(63)	0.13
	B	GLI3- FL/R; $Grk2^{+/+}$ + SAG vs. $Grk2^{-/-}$ + SAG	Mann-Whitney U test	11	0.0004
	B	GLI1; $Grk2^{+/+}$ + SAG vs. $Grk2^{-/-}$ + SAG	Mann-Whitney U test	11	0.0001
	C	Control vs. CMPD101	Mann-Whitney U test	60(63)	8.2 x 10-10
		Control + SAG vs. CMPD101 + SAG	Mann-Whitney U test	63(65)	0.13
	D	GLI3- FL/R; Control + SAG vs. CMPD101 + SAG	Mann-Whitney U test	9	0.0272
	D	GLI1; Control + SAG vs. CMPD101 + SAG	Mann-Whitney U test	9	0.0004
	E	Control + SAG vs. Paroxetine + SAG	Mann-Whitney U test	59(68)	0.88
	F	GLI3- FL/R; Control + SAG vs. Paroxetine + SAG	Mann-Whitney U test	7	0.0175
	F	GLI1; Control + SAG vs. Proxetine + SAG	Mann-Whitney U test	9	0.0019
	G	$Grk2^{+/+}$ + SAG vs. $Grk2^{-/-}$ + SAG	Mann-Whitney U test	68(69)	1.1 x 10-9
	H	GLI3- FL/R; $Grk2^{+/+}$ + SAG vs. $Grk2^{-/-}$ + SAG	Mann-Whitney U test	9	0.0399
	H	GLI1; $Grk2^{+/+}$ + SAG vs. $Grk2^{-/-}$ + SAG	Mann-Whitney U test	5	0.0159

	I	shScr + Dox vs. shGRK2 + SAG	Mann-Whitney U test	6	0.0028
	J	shSCR + SAG + Dox vs. shGRK2 + SAG + Dox	Mann-Whitney U test	65(66)	0.92
	K	Grk2$^{+/+}$ + SMO vs. Grk2$^{-/-}$ + SMO	Welch's *t*-test	3	0.0115
		Grk2$^{+/+}$ + SMO vs. Grk2$^{+/+}$+ SMO + SAG	Welch's *t*-test	3	0.13
		Grk2$^{+/+}$ + SMO + SAG vs. Grk2$^{-/-}$ + SMO + SAG	Welch's *t*-test	3	0.0051
		Grk2$^{+/+}$ + SMO + GRK2 vs. Grk2$^{-/-}$ + SMO + GRK2	Welch's *t*-test	3	0.75
		Grk2$^{+/+}$ + SMO + GRK2 vs. Grk2$^{+/+}$+ SMO + GRK2 + SAG	Welch's *t*-test	3	0.0804
		Grk2$^{+/+}$ + SMO + GRK2 + SAG vs. Grk2$^{-/-}$ + SMO + GRK2 + SAG	Welch's *t*-test	3	0.99
EV4	A	Control + Wnt3A + RSOP1 vs. Paroxetine + Wnt3A + RSPO1	Mann-Whitney U test	4	0.021
	B	LRP6/Actin; Control + Wnt3A + RSOP1 vs. Paroxetine + Wnt3A + RSPO1	Welch's *t*-test	3	0.0006
	B	pLRP6^{S1490}/Actin; Control + Wnt3A + RSOP1 vs. Paroxetine + Wnt3A + RSPO1	Welch's *t*-test	3	0.1
	B	pDVL2/DVL2; Control + Wnt3A + RSOP1 vs. Paroxetine + Wnt3A + RSPO1	Welch's *t*-test	3	0.042
	C	Control vs. Paroxetine	Welch's *t*-test	3	0.044
		Control + Wnt3A vs. Paroxetine + Wnt3A	Welch's *t*-test	3	0.006
	C	pLRP6^{S1490}/Actin; Control + Wnt3A vs. Paroxetine + Wnt3A	Welch's *t*-test	3	0.032
	C	pDVL2/DVL2; Control + Wnt3A vs. Paroxetine + Wnt3A	Welch's *t*-test	3	0.005
	D	Control vs. Paroxetine	Mann-Whitney U test	4	0.028
		Control + Wnt3A vs. Paroxetine + Wnt3A	Mann-Whitney U test	4	0.021
	D	pLRP6^{S1490}/Actin; Control + Wnt3A vs. Paroxetine + Wnt3A	Mann-Whitney U test	4	0.021
	D	pDVL2/DVL2; Control + Wnt3A vs. Paroxetine + Wnt3A	Mann-Whitney U test	4	0.021
	E	Control vs. Paroxetine	Mann-Whitney U test	4	0.029
		Control + Wnt3A vs. Paroxetine + Wnt3A	Mann-Whitney U test	4	0.28
	E	pLRP6^{S1490}/Actin; Control + Wnt3A vs. Paroxetine + Wnt3A	Mann-Whitney U test	4	0.021
	E	pDVL2/DVL2; Control + Wnt3A vs. Paroxetine + Wnt3A	Mann-Whitney U test	4	0.021
EV5		Control vs. R05-365A	Mann-Whitney U test	4	0.88
EV5		Grk2$^{+/+}$ vs. Grk2$^{-/-}$	Mann-Whitney U test	6	0.35
