# Supplementary material for: Mutations in GRK2 cause Jeune syndrome by impairing Hedgehog and canonical Wnt signaling
Source: EMBO Mol Med. 2020 Oct 14;12(11):e11739. doi: 10.15252/emmm.201911739 (PMC7645380; doi:10.15252/emmm.201911739)
Supplement: Supplementary file 2 — Expanded View Figures PDF [file EMMM-12-e11739-s002.pdf]

Expanded View Figures

Figure EV1. GRK2 is not required for ciliogenesis or IFT.

A–C Primary cilia are normal in *GRK2*$^{-/-}$ fibroblasts. Cells were serum starved for 24 or 48 h to induce cilia, and (A) immunostained for acetylated tubulin (AcTu, red), ARL13B (green) (upper panel; insets show the individual signals), or AcTu (red), pericentrin (red) and GLI3 (green) (lower panel). Control and *GRK2*$^{-/-}$ cilia were positive for AcTu and ARL13B, and showed similar localization of GLI3 to the tips (lower panel, arrows). Acetylated tubulin and pericentrin staining were used to visualize the axoneme and centrioles, respectively. Scale bar, 2 μm. (B) Length distribution of cilia did not differ between control and *GRK2*$^{-/-}$ cells. Red bars show medians; dots represent individual cilia. Mann–Whitney *U* test; number of biological experiments and the total numbers of analyzed cilia are indicated. (C) There was no difference in the efficiency of ciliogenesis between control and *GRK2*$^{-/-}$ cells. Mean ± SEM. Welch's *t*-test; number of biological experiments and the total numbers of analyzed cells are indicated.

D Loss of GRK2 did not affect localization of ciliary and IFT components. Immunofluorescence of serum-starved (48 hr) control and *GRK2*$^{-/-}$ fibroblasts immunostained with AcTu or detyrosinated tubulin (DetyrTu) to mark the cilium (red), and IFT43, IFT88, KIF3A, TRAF3IP1, WDR34, or ICK (green). No significant differences in staining were found between control and *GRK2*$^{-/-}$ cilia, suggesting normal IFT. Scale bars, 5 μm and 1 μm (insets).

E–G Inhibition of GRK2 activity did not affect primary cilia in chondrocytes. (E) Control human R92-284 and R00-082 chondrocytes were serum starved in the presence of a GRK2 inhibitor, either 20 μM CMPD101 or 10 μM paroxetine, for 24 h before they were fixed and immunostained for cilia (ARL13B; gamma tubulin, γTu; acetylated tubulin, AcTu) and the retrograde IFT-B proteins IFT81 or IFT88. Scale bar, 1 μm. (F) Cilia length was measured using the ARL13B signal. Red bar, median. Mann–Whitney *U*-test; number of biological experiments and total cilia numbers are indicated. (G) The percentage of ciliated cells was calculated. Mean ± SEM. Welch's *t*-test; number of biological experiments and total cell numbers are indicated.

Source data are available online for this figure.

© 2020 The Authors

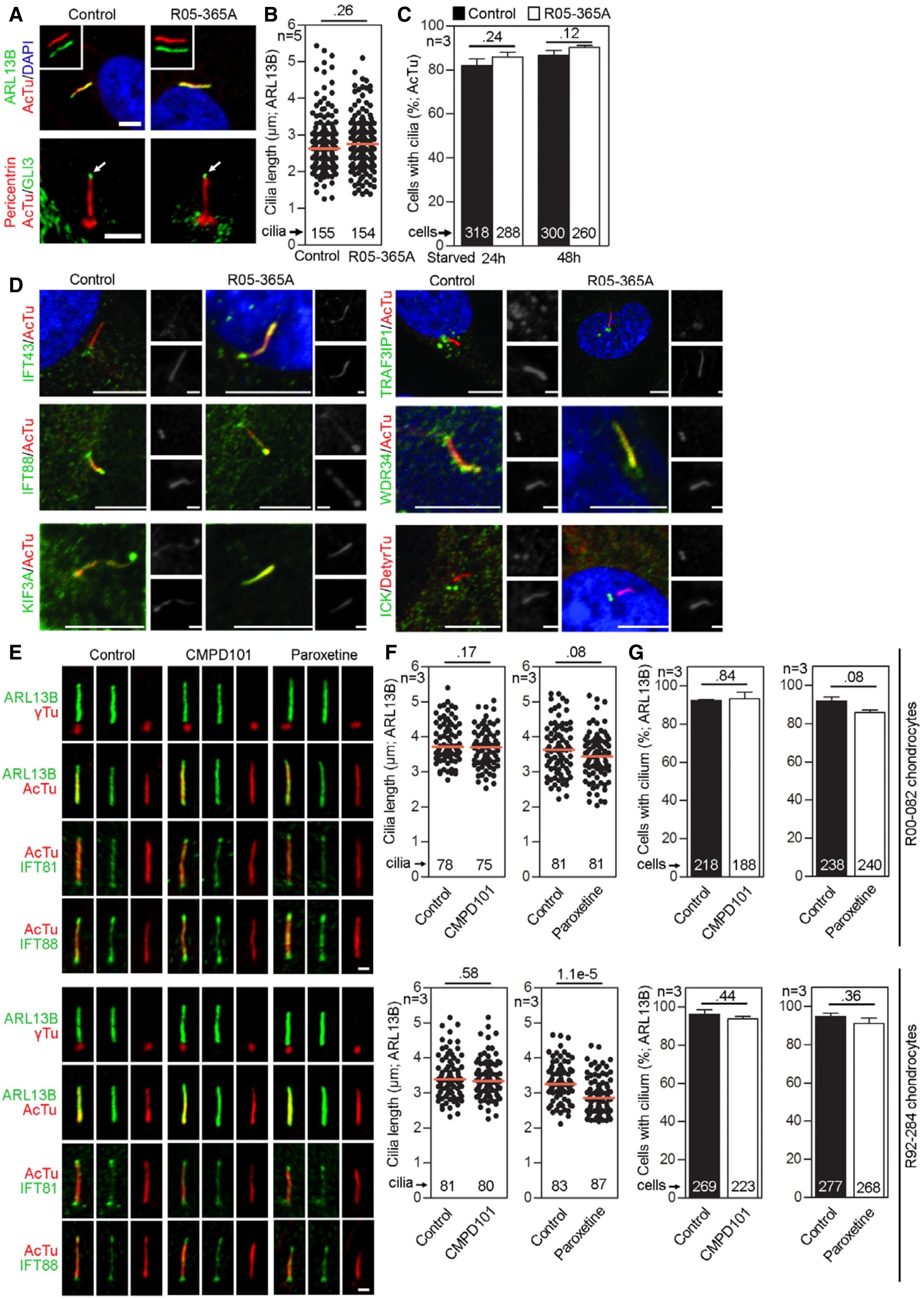

Figure EV1.

 © 2020 The Authors

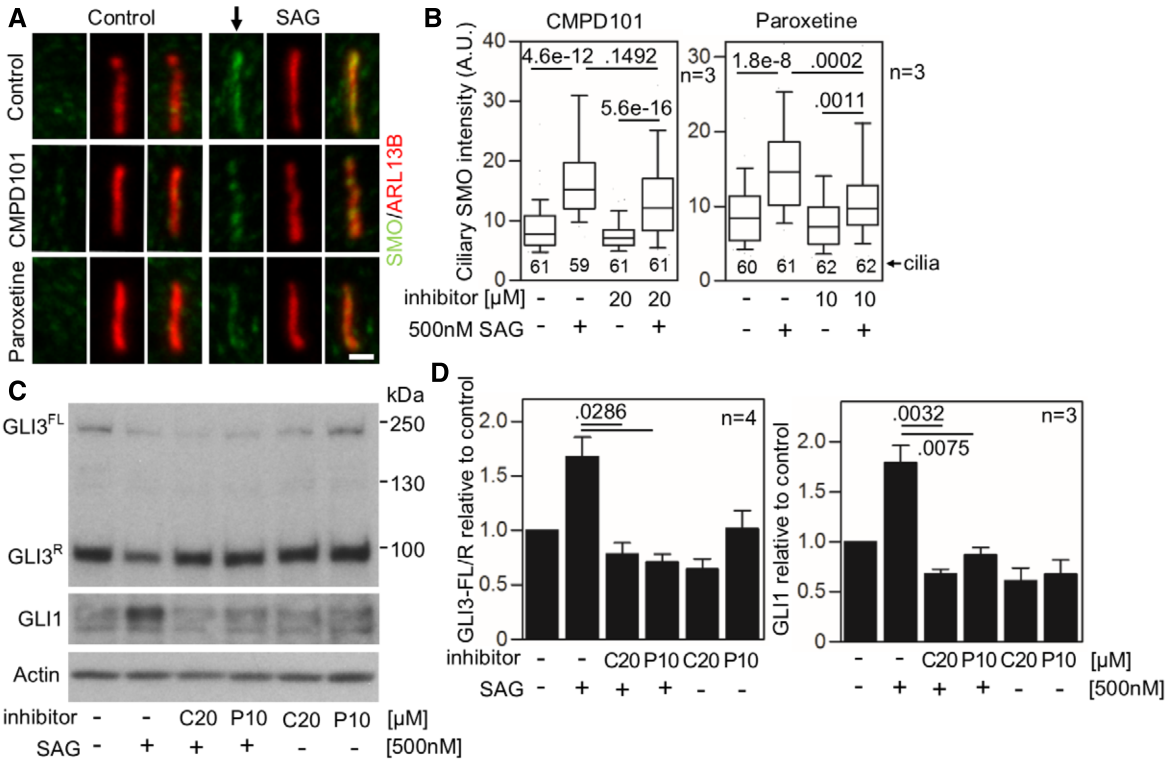

Figure EV2. Inhibition of GRK2 activity decreases SMO accumulation in cilia and GLI activity in the human chondrocytes.

A–D Defective SMO cilia accumulation and inhibited Hh signaling in chondrocytes treated with GRK2 inhibitors. Control human R92-284 chondrocytes were serum starved in the presence of a GRK2 inhibitor, either CMPD101 (C) or paroxetine (P), for 24 h before they were treated with SAG for additional 12 (A, B) or 24 h (C, D). (A, B) Cilia were stained by ARL13B and SMO antibodies, and the intensity of ciliary SMO was analyzed and plotted. Note the impaired SMO accumulation in cilia caused by GRK2 inhibition (black arrow). Central band, median. Box, 1st–3rd quartile. Whiskers, 10%–90% percentile. Mann–Whitney *U*-test; number of biological experiments and the total numbers of analyzed cilia are indicated. Scale bar, 1 μm. (C) Cell lysates were immunoblotted for GLI3 processing and GLI1 upregulation; actin levels served as a loading control. GLI3^{FL} and GLI3^{R}, full-length and repressor GLI3 variants, respectively. (D) Quantification of GLI3 and GLI1 levels by densitometry. Note the impaired GLI3 processing and no GLI1 upregulation in cells treated with CMPD101 or paroxetine. Mean ± SEM. Mann–Whitney *U*-test (GLI3 ratio) and Welch's *t*-test (GLI1 levels); number of biological experiments is indicated.

Source data are available online for this figure.

Figure EV3. Loss of Grk2 protein or its activity does not prevent ciliary SMO accumulation in the murine NIH3T3 cells.

A Serum-starved *Grk2*$^{+/+}$ and *Grk2*$^{-/-}$ NIH3T3 cells were treated with 500 nM SAG for 4 h; the cells were immunostained for ARL13B and SMO, and the intensity of ciliary SMO was analyzed and plotted. Note the normal ciliary SMO intensity in *Grk2*$^{-/-}$ cells. Central band, median. Box, 1st–3rd quartile. Whiskers, 10–90% percentile. Mann–Whitney *U* test; number of biological experiments and the total numbers of analyzed cilia are indicated. Scale bars, 1 μm.

B Serum-starved *Grk2*$^{+/+}$ and *Grk2*$^{-/-}$ NIH3T3 cells were treated with 500 nM SAG for 10–16 h, and immunoblotted for Gli3 and Gli1. The optical density of the bands was normalized to that of actin, and the Gli3-FL/R ratios and Gli1 protein levels were plotted. Mean ± SEM. Mann–Whitney *U*-test; number of biological experiments is indicated. Gli3^{FL} and Gli3^{R}, full-length and repressor GLI3 variants, respectively.

C–F *Grk2*$^{+/+}$ NIH3T3 cells were serum starved together with 25 μM CMPD101 (C, D) or 1 μM paroxetine (E, F) for 12 h, and treated with 500 nM SAG for 4 hours (C, E) or 12 h (D, F). (C, E) Inhibition of Grk2 activity by neither CMPD101 nor paroxetine prevents the SAG-induced ciliary SMO accumulation. Central band, median. Box, 1st–3rd quartile. Whiskers, 10%–90% percentile. Mann–Whitney *U* test; number of biological experiments and the total numbers of analyzed cilia are indicated. Scale bars, 1 μm. (D, F) CMPD101 and paroxetine inhibit SAG-mediated Gli3 processing and Gli1 upregulation. Mean ± SEM. Mann–Whitney *U* test; number of biological experiments is indicated.

G, H Micromasses derived from *Grk2*$^{+/+}$ and *Grk2*$^{-/-}$ NIH3T3 cells were serum starved, treated, and analyzed as in (C–F). (G) Note the inhibition of ciliary SMO intensity in *Grk2*$^{-/-}$ micromasses. Central band, median. Box, 1st–3rd quartile. Whiskers, 10%–90% percentile. Mann–Whitney *U*-test; number of biological experiments and the total numbers of analyzed cilia are indicated. Scale bars, 1 μm. (H) Western blots for Gli3 processing and Gli1 upregulation. Mean ± SEM. Mann–Whitney *U*-test; number of biological experiments is indicated. Vinc., vinculin.

I, J Normal SMO cilia accumulation in murine IMCD3 cells with downregulated Grk2. (I) Doxycycline (DOX)-inducible Grk2 downregulation, tested by Western blot, normalized to actin and plotted below. Mean ± SEM. Mann–Whitney *U*-test; number of biological experiments is indicated. shScr, scramble shRNA. (J) Cells pre-treated with DOX for 3 days were serum starved, SAG treated, stained, and analyzed as in (A), and the intensity of ciliary SMO was analyzed and plotted. Note the normal SMO accumulation in cilia in cells with Grk2 downregulation. Central band, median. Box, 1st–3rd quartile. Whiskers, 10%–90% percentile. Mann–Whitney –test; number of biological experiments and the total numbers of analyzed cilia are indicated. Scale bar, 1 μm.

K Under-phosphorylation of SMO in *Grk2*$^{-/-}$ NIH3T3 cells. Lysates of SMO-transfected cells were resolved by the phospho(p)-shift PAGE and immunoblotted for SMO. The portion of pSMO analyzed by densitometry is shown. GRK2 add-back demonstrates a rescue of the pSMO in *Grk2*$^{-/-}$ cells. Mean ± SEM. Welch's *t*-test; number of biological replicates is indicated.

Source data are available online for this figure.

 © 2020 The Authors

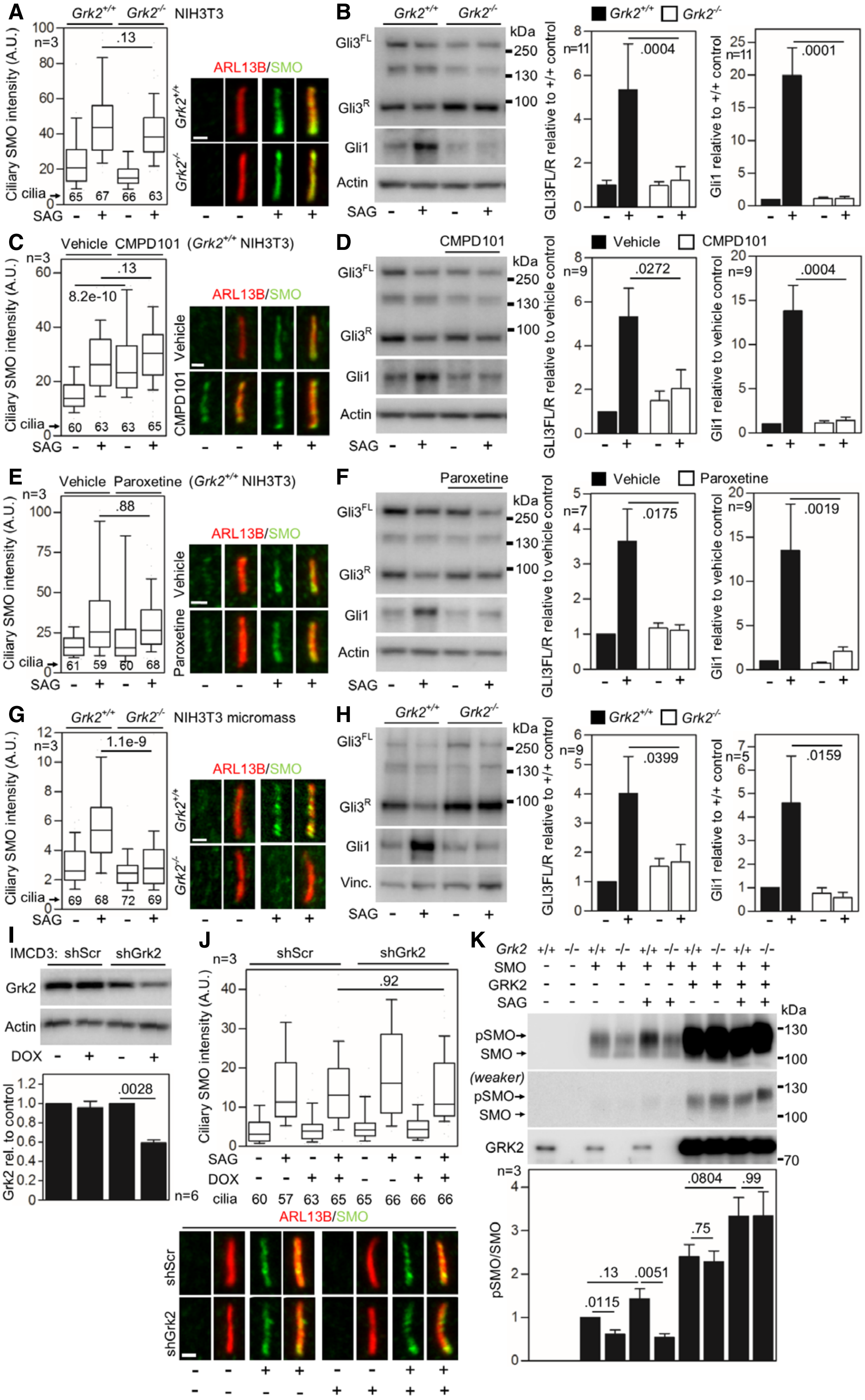
A
Grk2+/+ Grk2-/- NIH3T3
Ciliary SMO intensity (A.U.)
ARL13B/SMO
B
GLI3FL/R relative to +/+ control
Gli1 relative to +/+ control
C
Vehicle CMPD101 (Grk2+/+ NIH3T3)
D
CMPD101
GLI3FL/R relative to vehicle control
Gli1 relative to vehicle control
E
Vehicle Paroxetine (Grk2+/+ NIH3T3)
F
Paroxetine
G
Grk2+/+ Grk2-/- NIH3T3 micromass
H
I
IMCD3: shScr shGrk2
Grk2 rel. to control
J
shScr shGrk2
K
pSMO/SMO

Figure EV3.

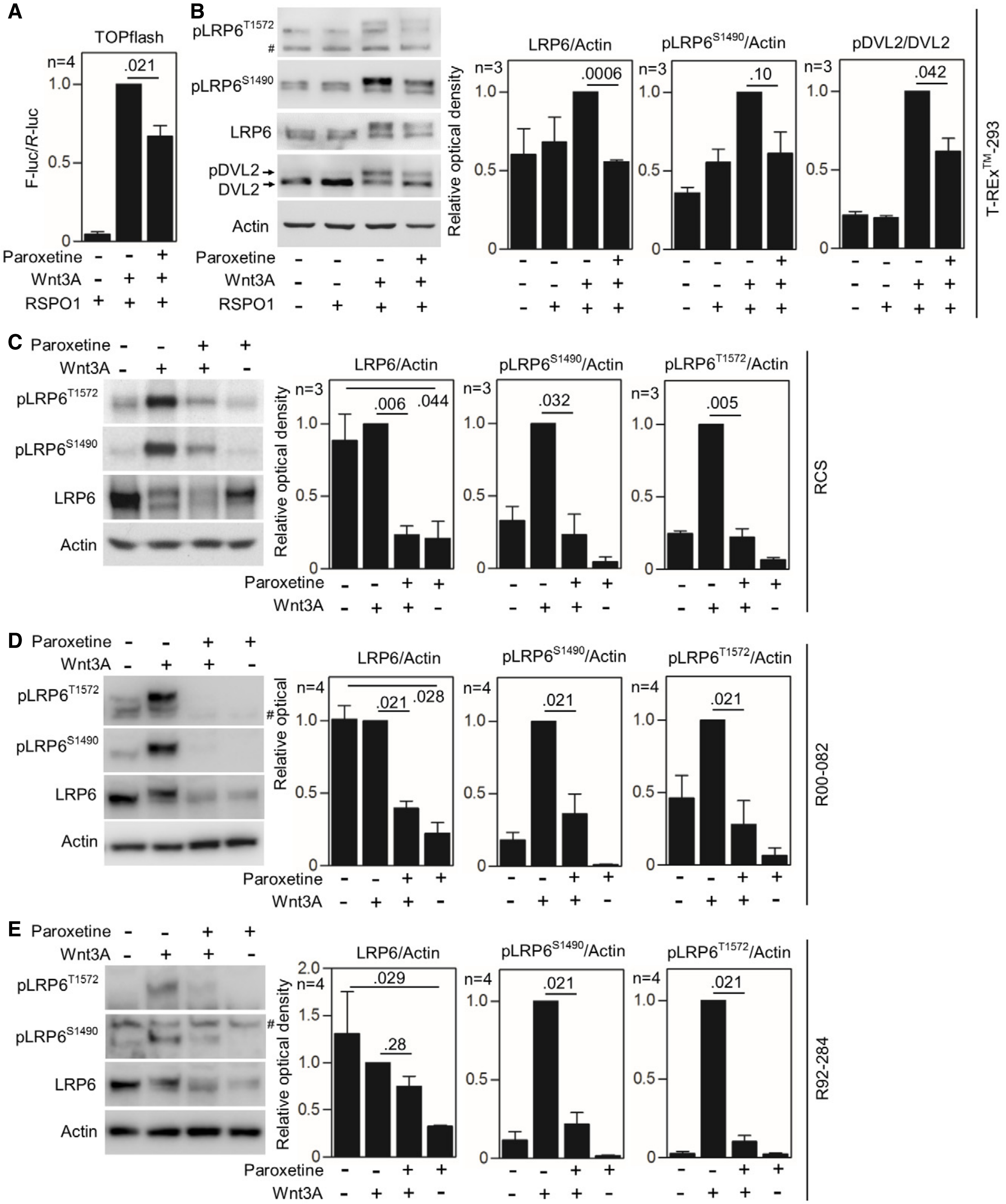
A
TOPflash
n=4
.021
F-luc/R-luc
Paroxetine
Wnt3A
RSPO1
B
pLRP6T1572
pLRP6S1490
LRP6
pDVL2
DVL2
Actin
LRP6/Actin
pLRP6S1490/Actin
pDVL2/DVL2
n=3
.0006
.10
.042
Relative optical density
T-REx™-293
C
LRP6/Actin
pLRP6S1490/Actin
pLRP6T1572/Actin
.006
.044
.032
.005
RCS
D
n=4
.021
.028
R00-082
E
.029
.28
R92-284

Figure EV4.

◀ **Figure EV4. Inhibition of GRK2 activity inhibits canonical Wnt signaling.**

A T-REx™-293 cells were transfected with the TOPflash Firefly (F) luciferase vector together with a control *Renilla* (R) luciferase vector, and the effect of Wnt3A on TOPflash transcriptional activation was determined by dual-luciferase assay. Note the less Wnt3A-induced TOPflash *trans*-activation in cells treated with 20 μM paroxetine. R-spondin-1 (RSPO1) was used to block endogenous inhibition of canonical Wnt. Mean ± SEM. Mann–Whitney *U*-test; number of biological experiments is indicated.

B T-REx™ 293 cells with GRK2 inhibited by 20 μM paroxetine show lower levels of LRP6 and lower pDVL2/DVL2 ratio, as determined by Western blot and quantified by densitometry. No significant Wnt3A-induced LRP6-T1572 phosphorylation was observed in T-REx™ 293 cells. Mean ± SEM. Welch's *t*-test; number of biological experiments is indicated.

C–E Rat chondrosarcoma (RCS) cells (C), and control R00-082 (D) and R92-284 (E) chondrocytes were treated with 20 μM paroxetine overnight and then treated with Wnt3A for 1 h. Note the lower levels of LRP6 and less LRP6 phosphorylation (pS1490- and pT1572-LRP6) in cells treated with paroxetine. #, nonspecific band. Mean ± SEM. Welch's *t*-test (C) and Mann–Whitney *U* test (D, E); number of biological experiments is indicated.

Source data are available online for this figure.

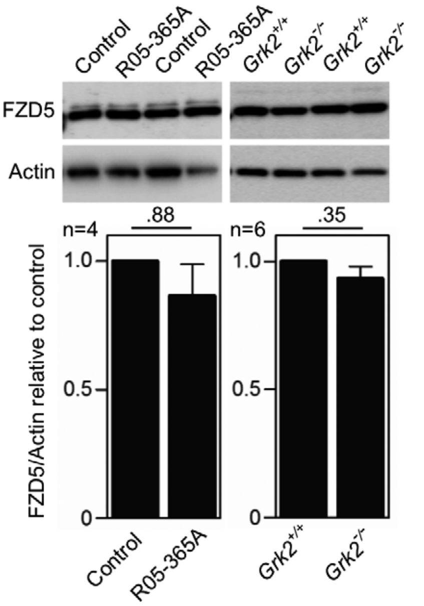

Figure EV5. Frizzled 5 (FZD5) levels are normal in R05-365A *GRK2*$^{-/-}$ fibroblasts and *Grk2*$^{-/-}$ NIH3T3 cells.

Lysates of R05-365A *GRK2*$^{-/-}$ fibroblasts and *Grk2*$^{-/-}$ NIH3T3 cells were analyzed by Western blots for expression levels of FZD5. After normalization to actin, the densitometry revealed no differences between the wild-type and GRK-null cells. Mean ± SEM. Mann–Whitney *U*-test; number of biological experiments is indicated.

Source data are available online for this figure.
