# Supplementary figures and images for: Mutations in GRK2 cause Jeune syndrome by impairing Hedgehog and canonical Wnt signaling
Source: EMBO Mol Med. 2020 Oct 14;12(11):e11739. doi: 10.15252/emmm.201911739 (PMC7645380; doi:10.15252/emmm.201911739)

Figure 2D

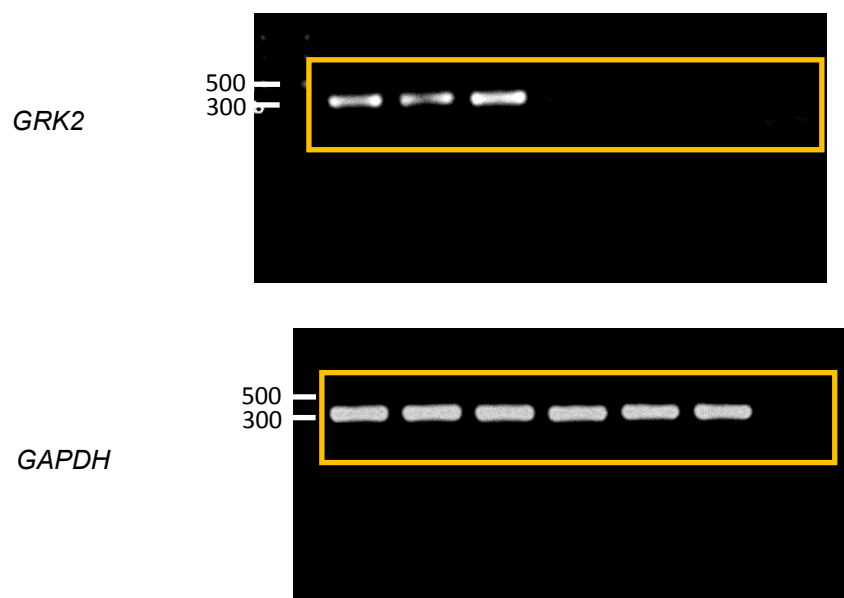

Figure 2E

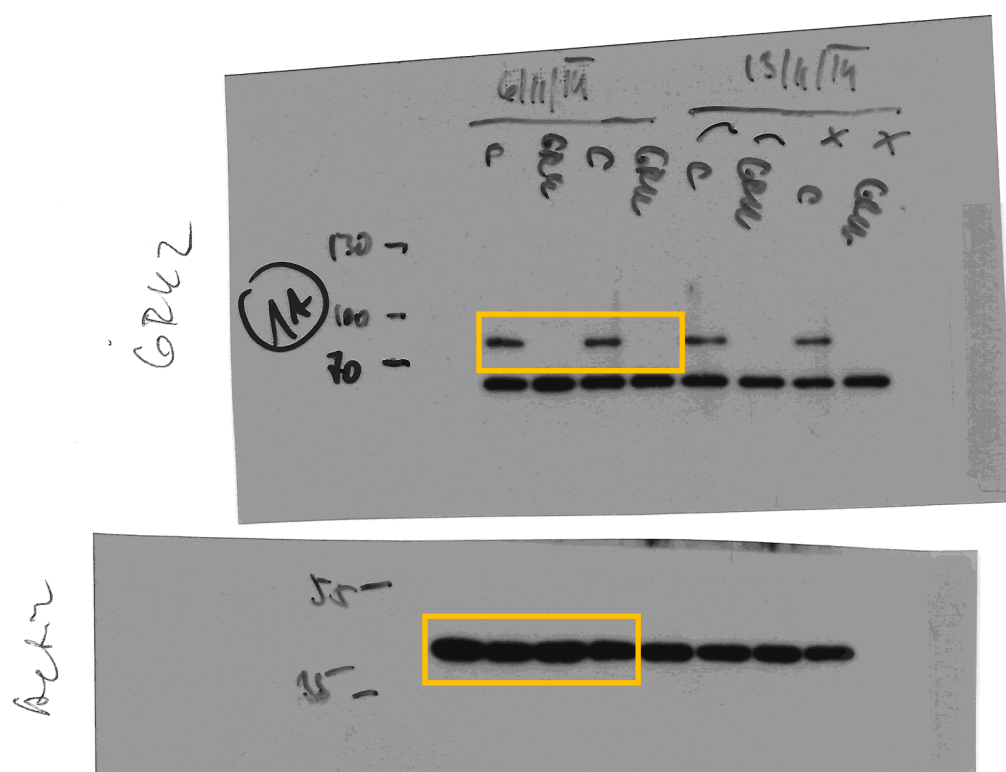

Supplement: Supplementary file 5 — Source Data for Figure 2 [file EMMM-12-e11739-s003.zip › EMM-2019-11739_SourceDataForFigure2/EMM-2019-11739_SourceDataForFigure2.pdf]

Figure 4A

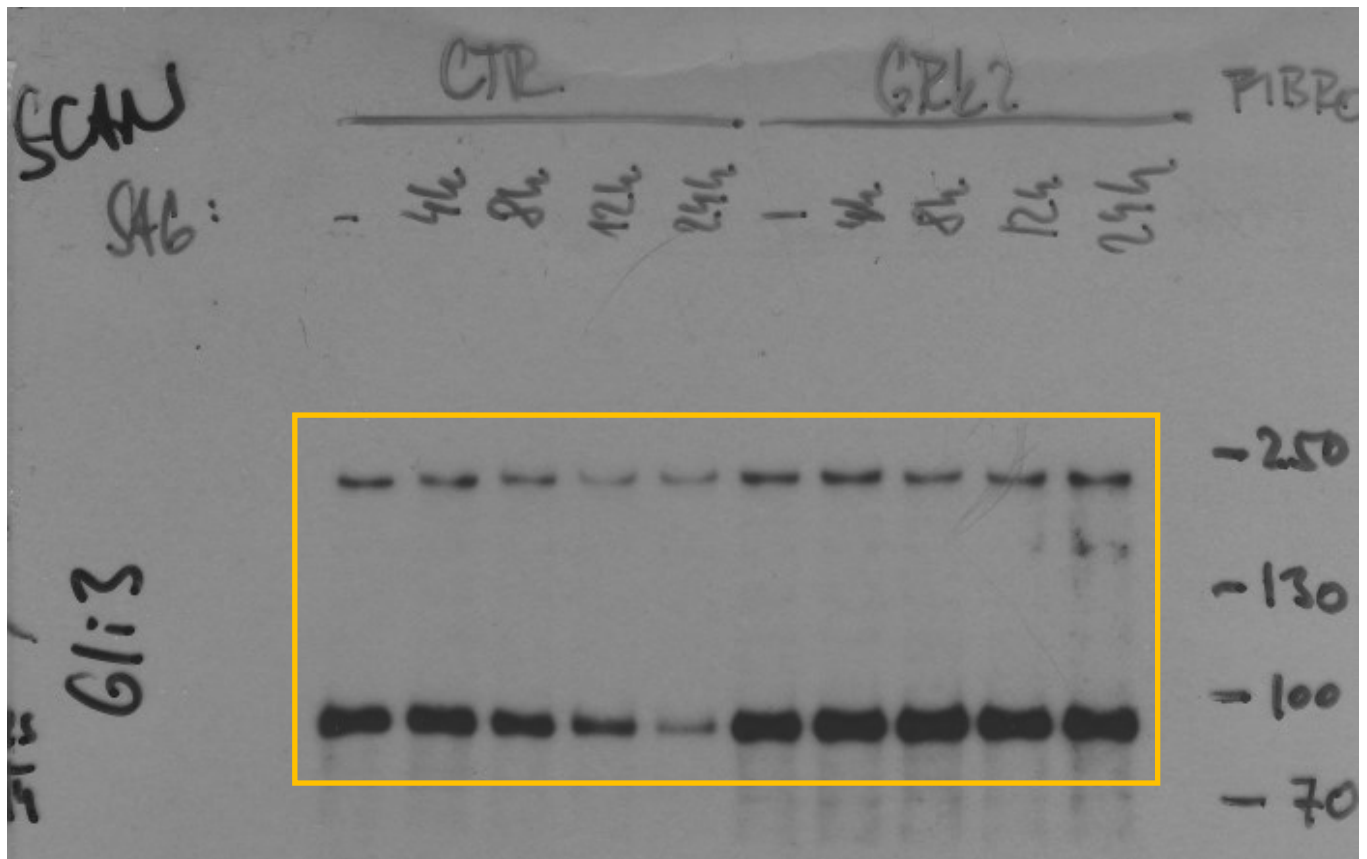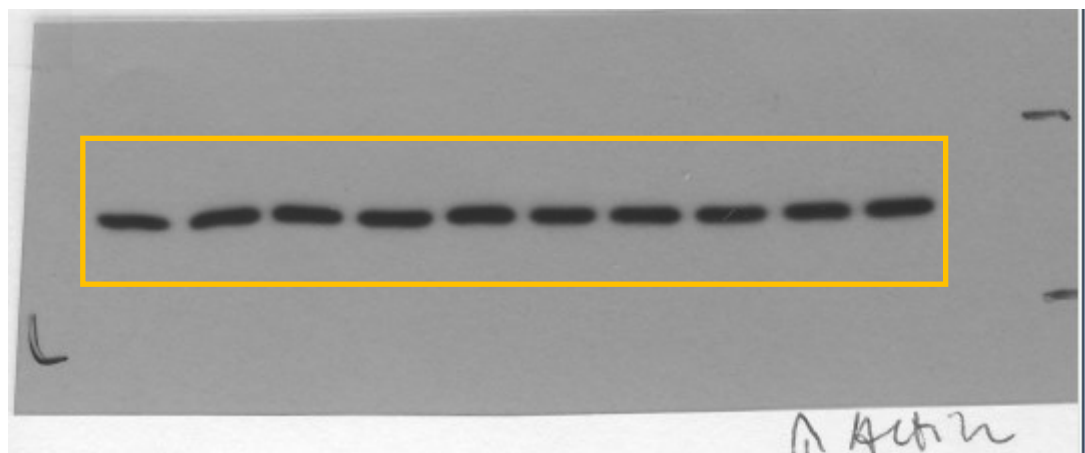

Figure 4D

SMO

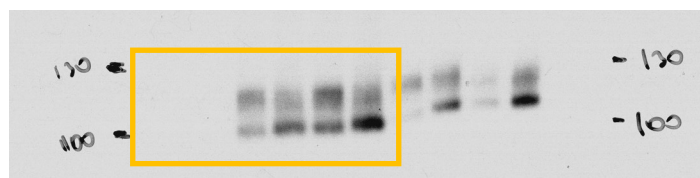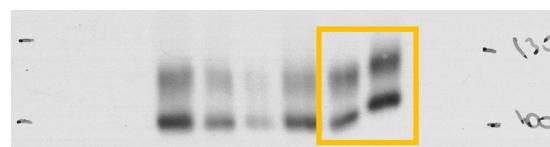

GRK2

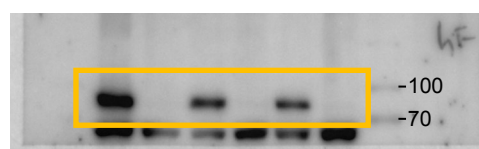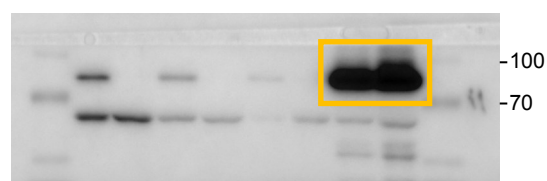

Figure 4E

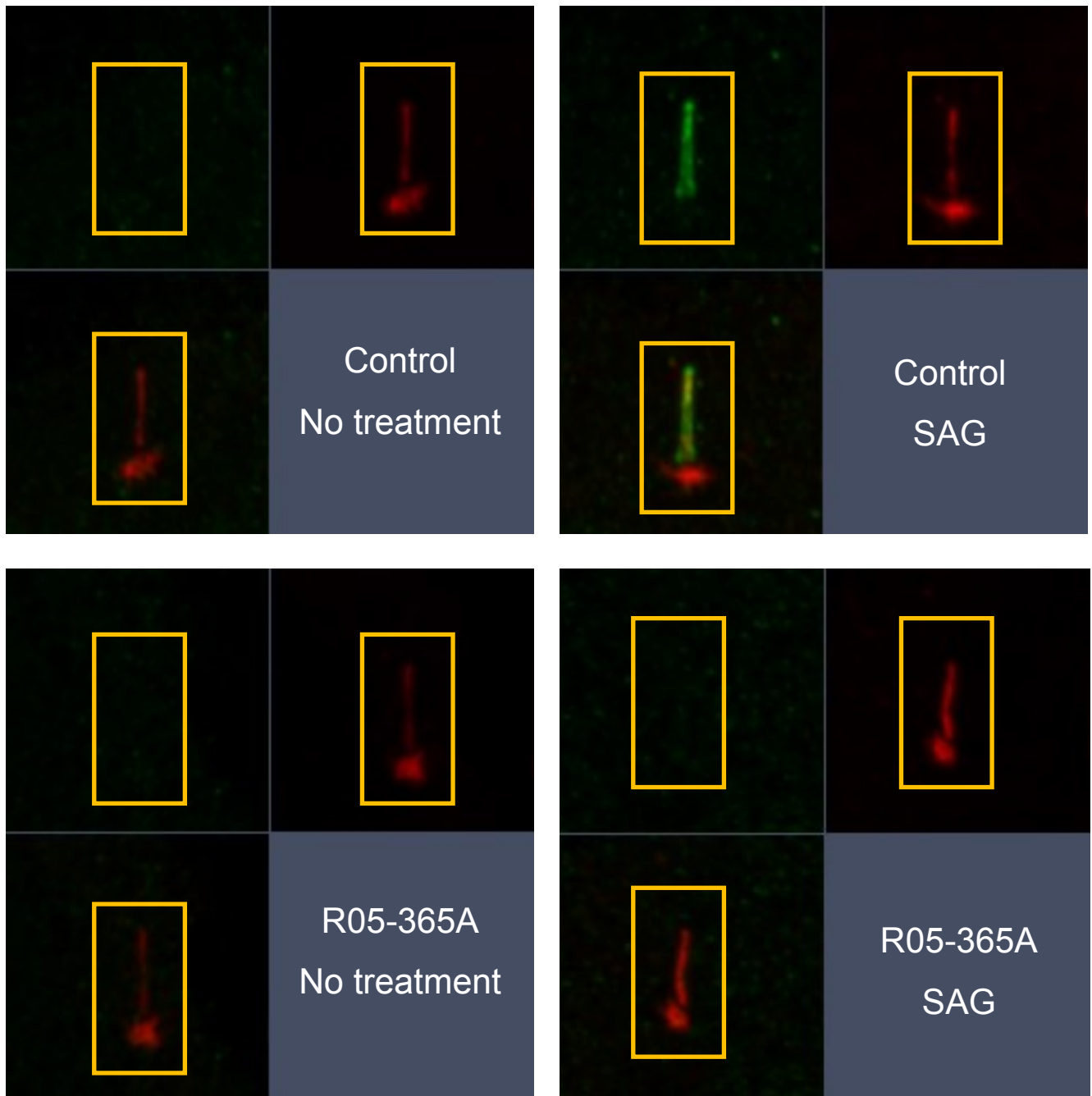

Supplement: Supplementary file 7 — Source Data for Figure 4 [file EMMM-12-e11739-s005.zip › EMM-2019-11739_SourceDataForFigure4/EMM-2019-11739_SourceDataForFigure4.pdf]

Figure 6B

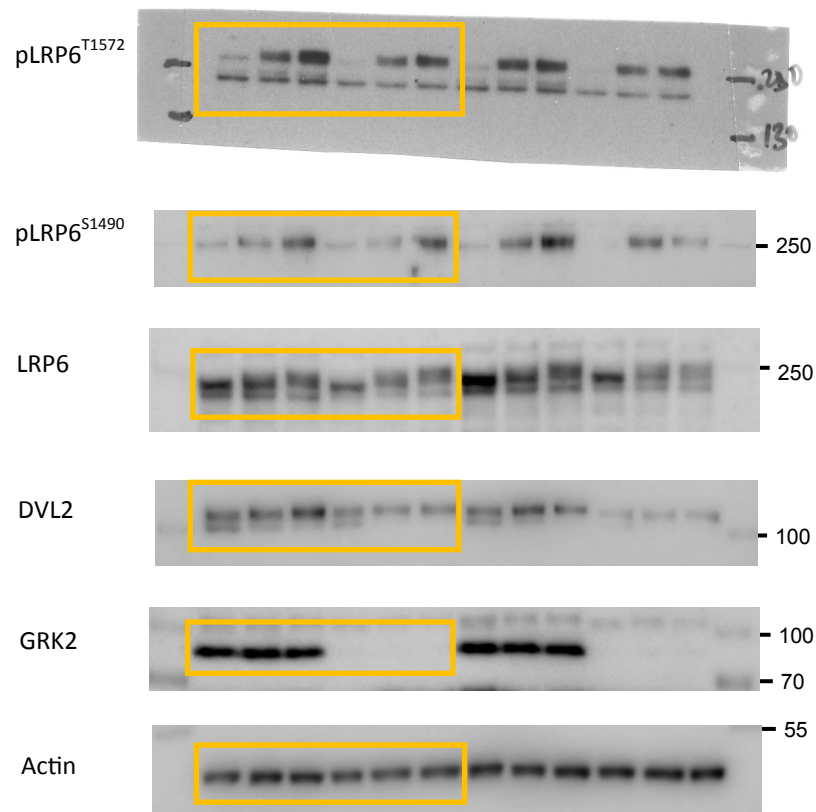

Figure 6D

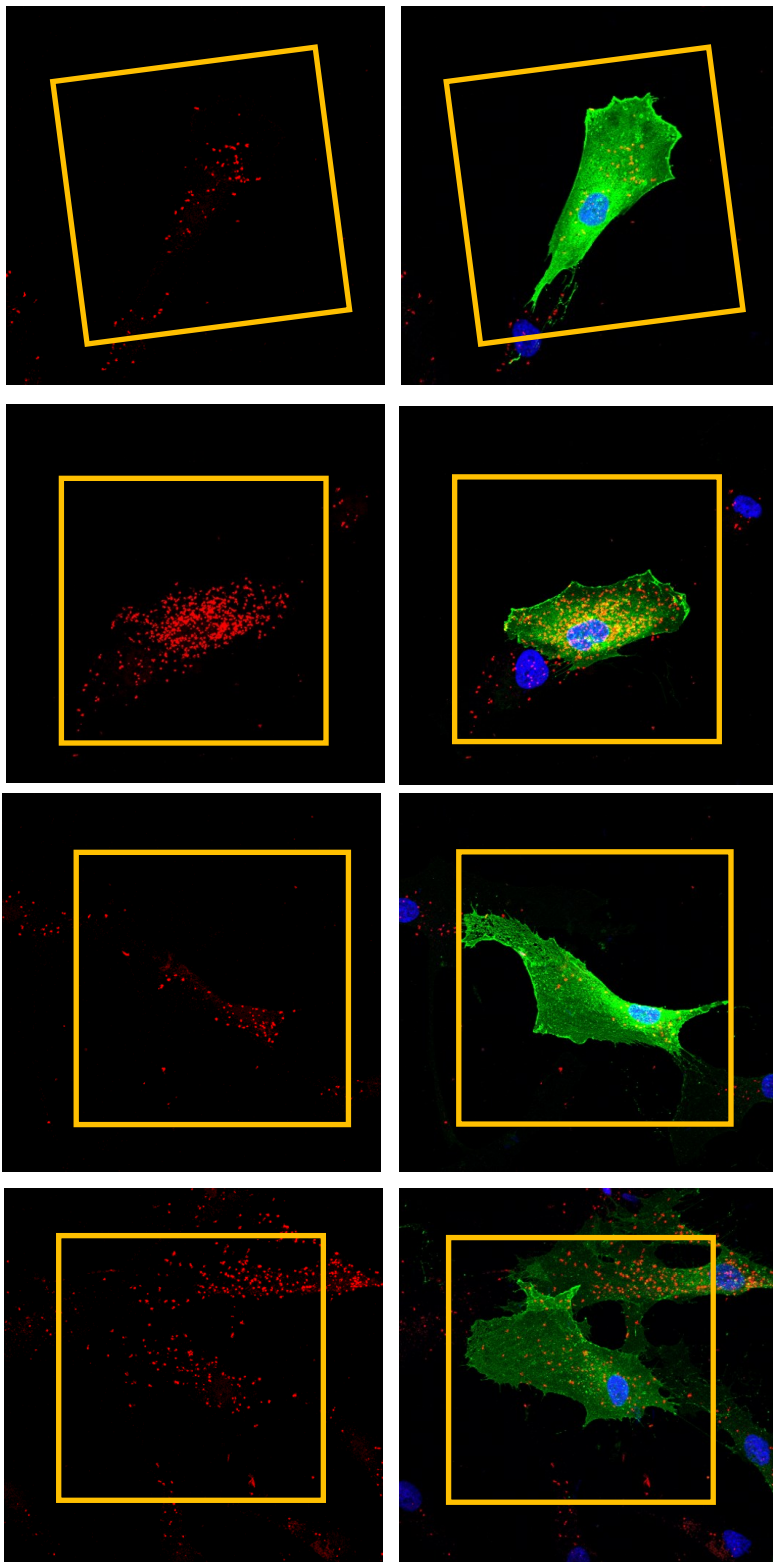

Supplement: Supplementary file 9 — Source Data for Figure 6 [file EMMM-12-e11739-s007.zip › EMM-2019-11739_SourceDataForFigure6/EMM-2019-11739_SourceDataForFigure6.pdf]

Figure 7B

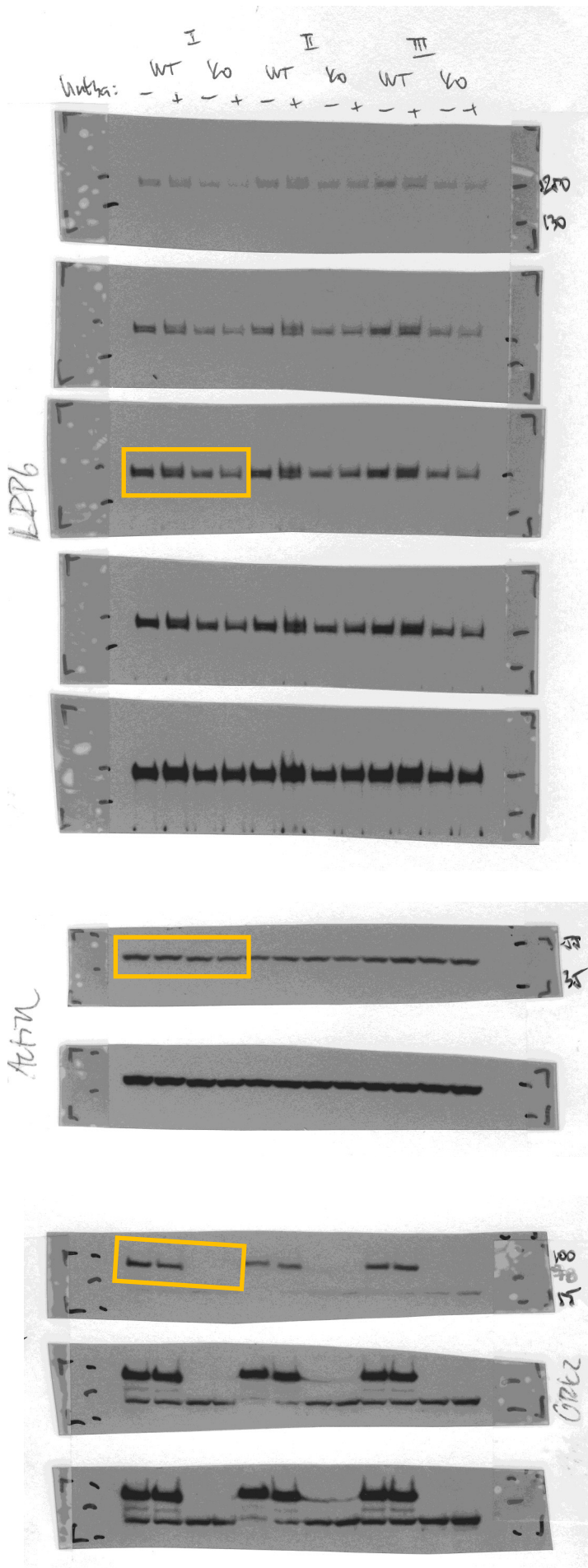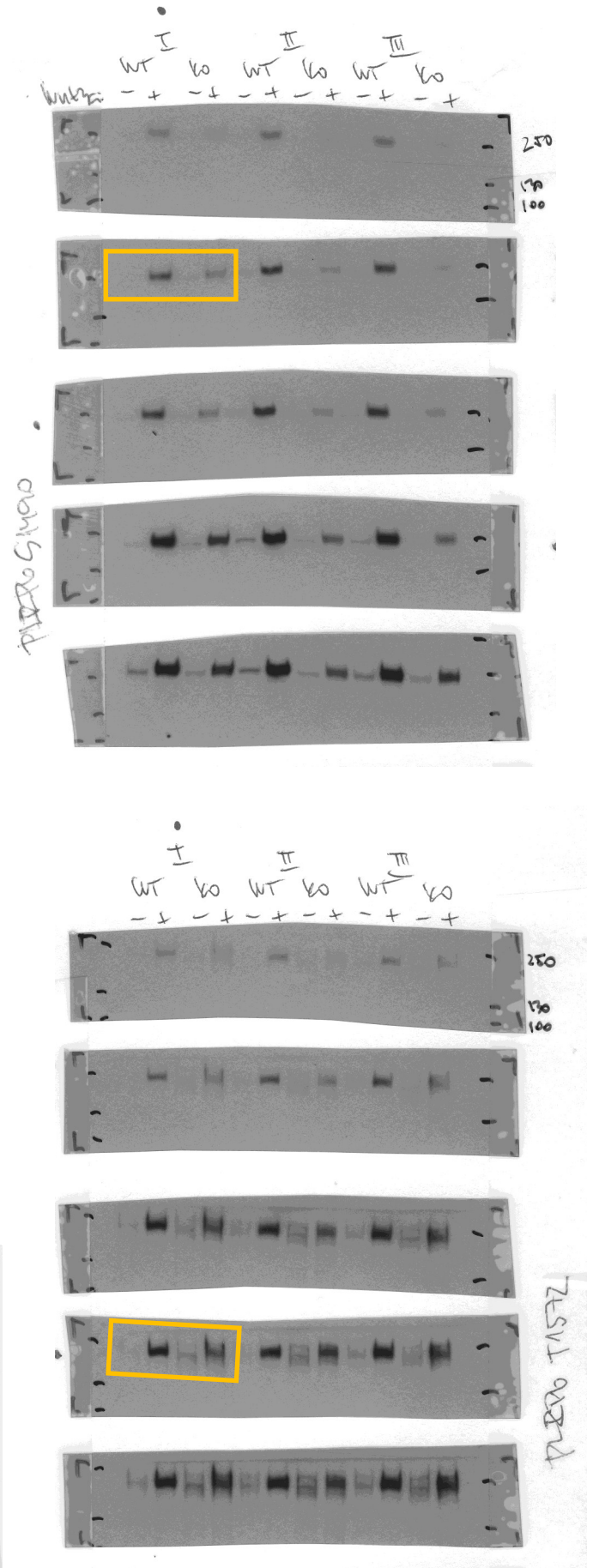

Figure 7C

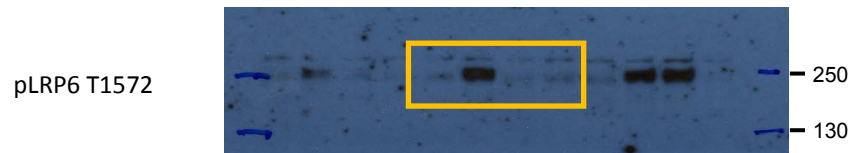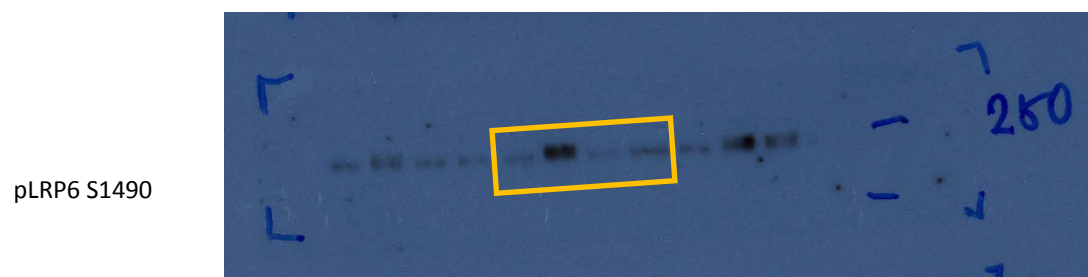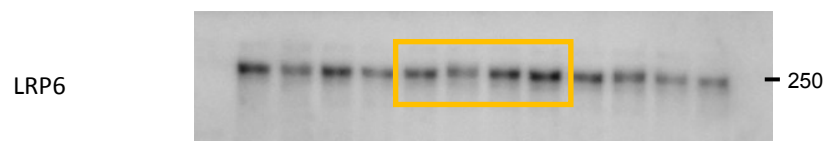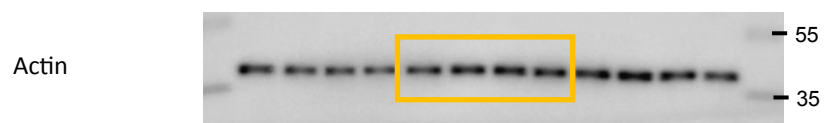

Figure 7D

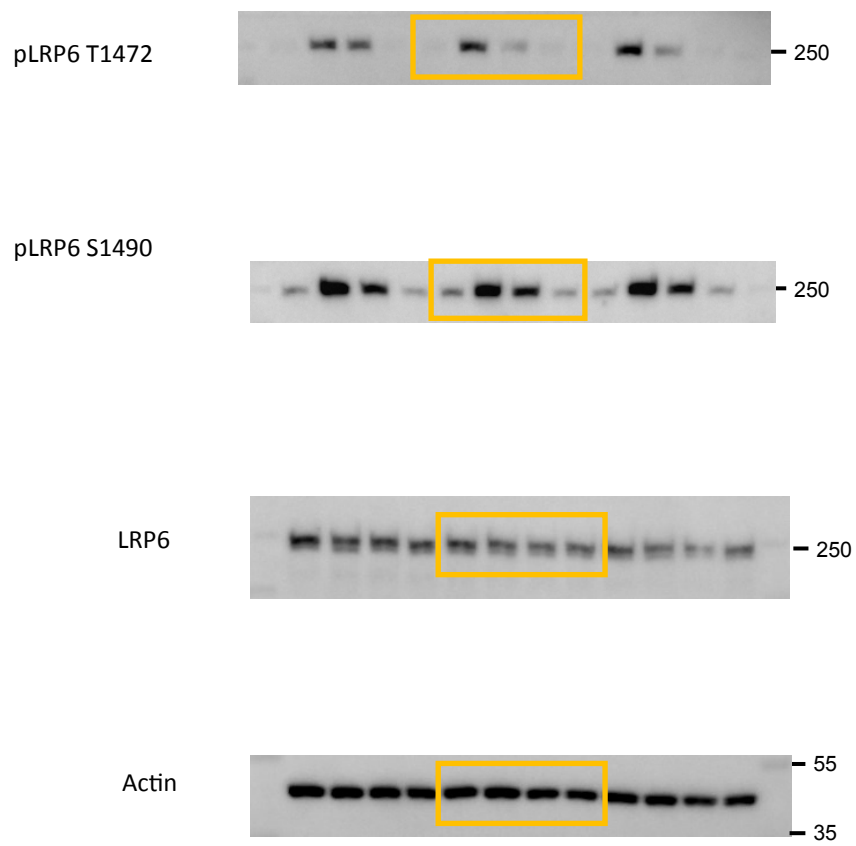

Figure 7E

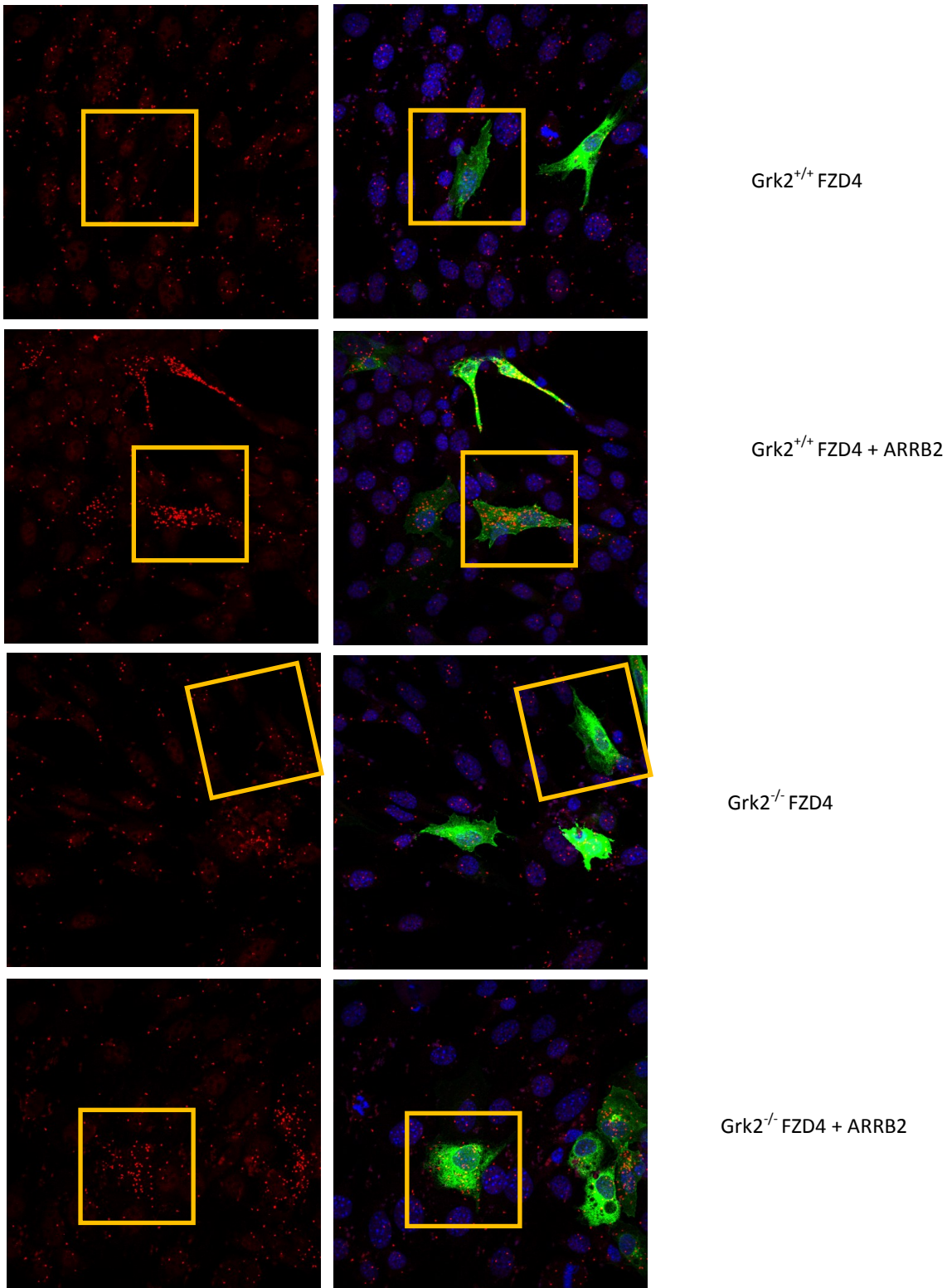

Figure 7F

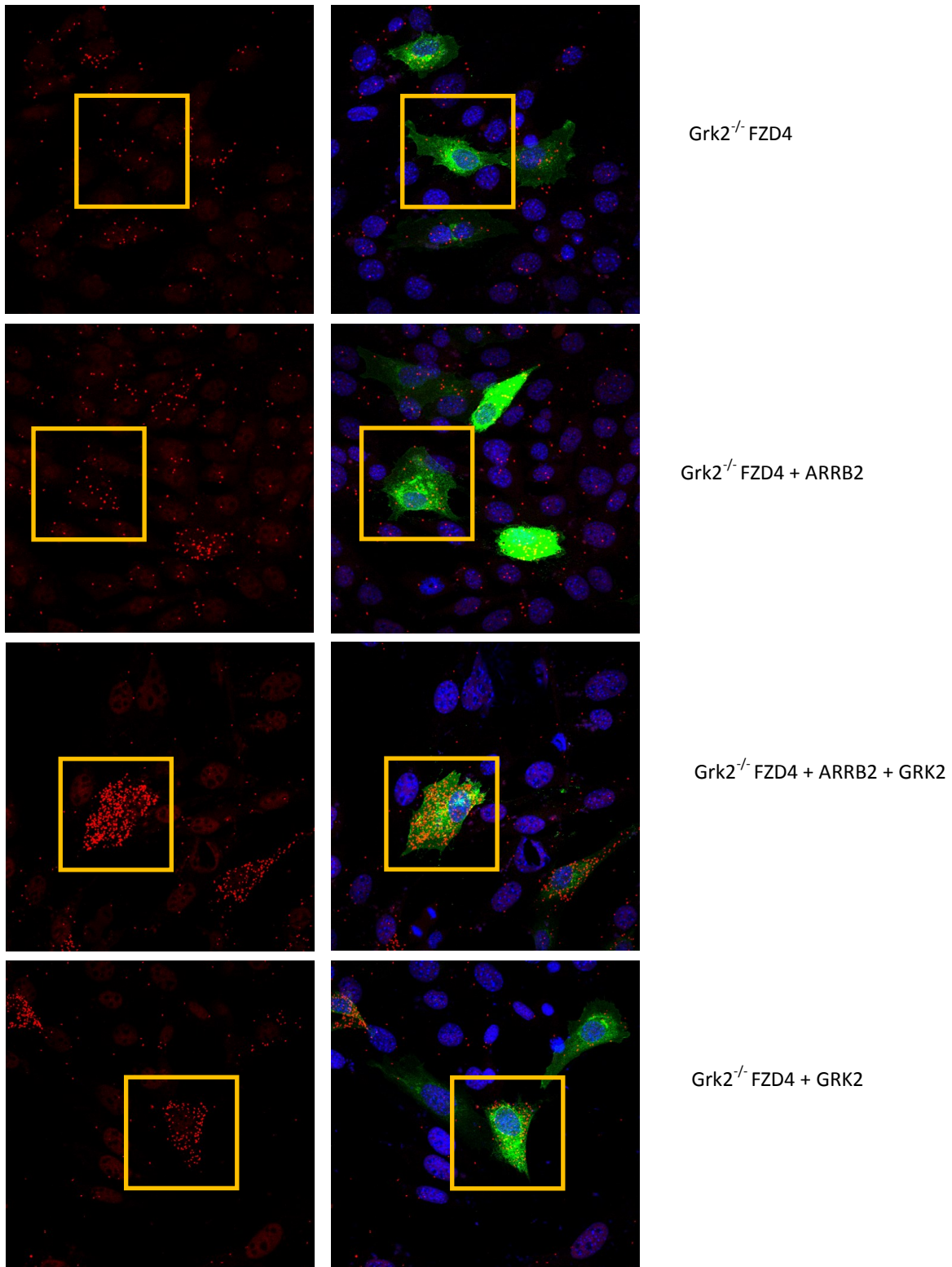

Supplement: Supplementary file 10 — Source Data for Figure 7 [file EMMM-12-e11739-s008.zip › EMM-2019-11739_SourceDataForFigure7/EMM-2019-11739_SourceDataForFigure7.pdf]
