# Supplementary material for: Mutations in GRK2 cause Jeune syndrome by impairing Hedgehog and canonical Wnt signaling
Source: EMBO Mol Med. 2020 Oct 14;12(11):e11739. doi: 10.15252/emmm.201911739 (PMC7645380; doi:10.15252/emmm.201911739)

Figure 3A

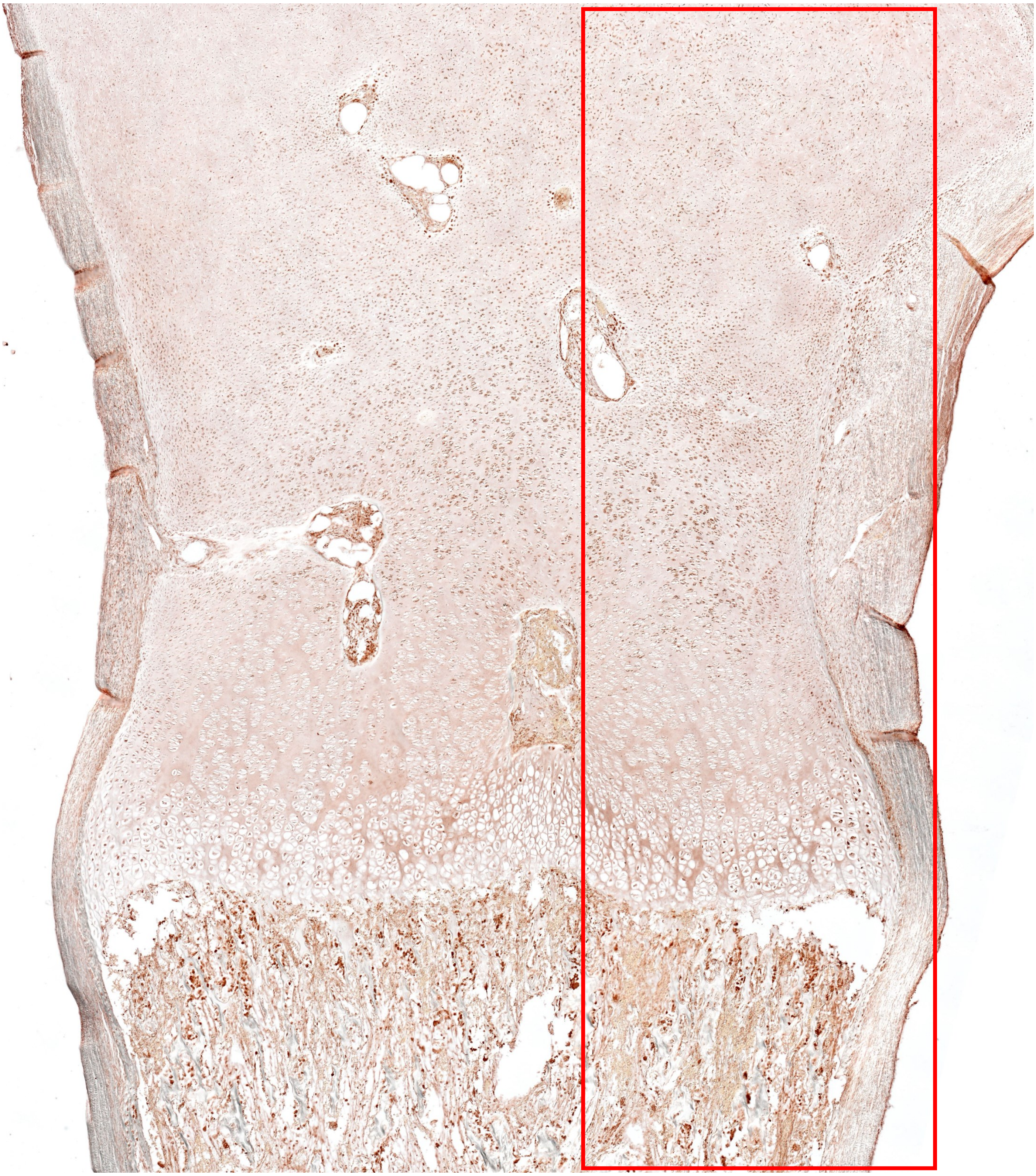

Figure 3C

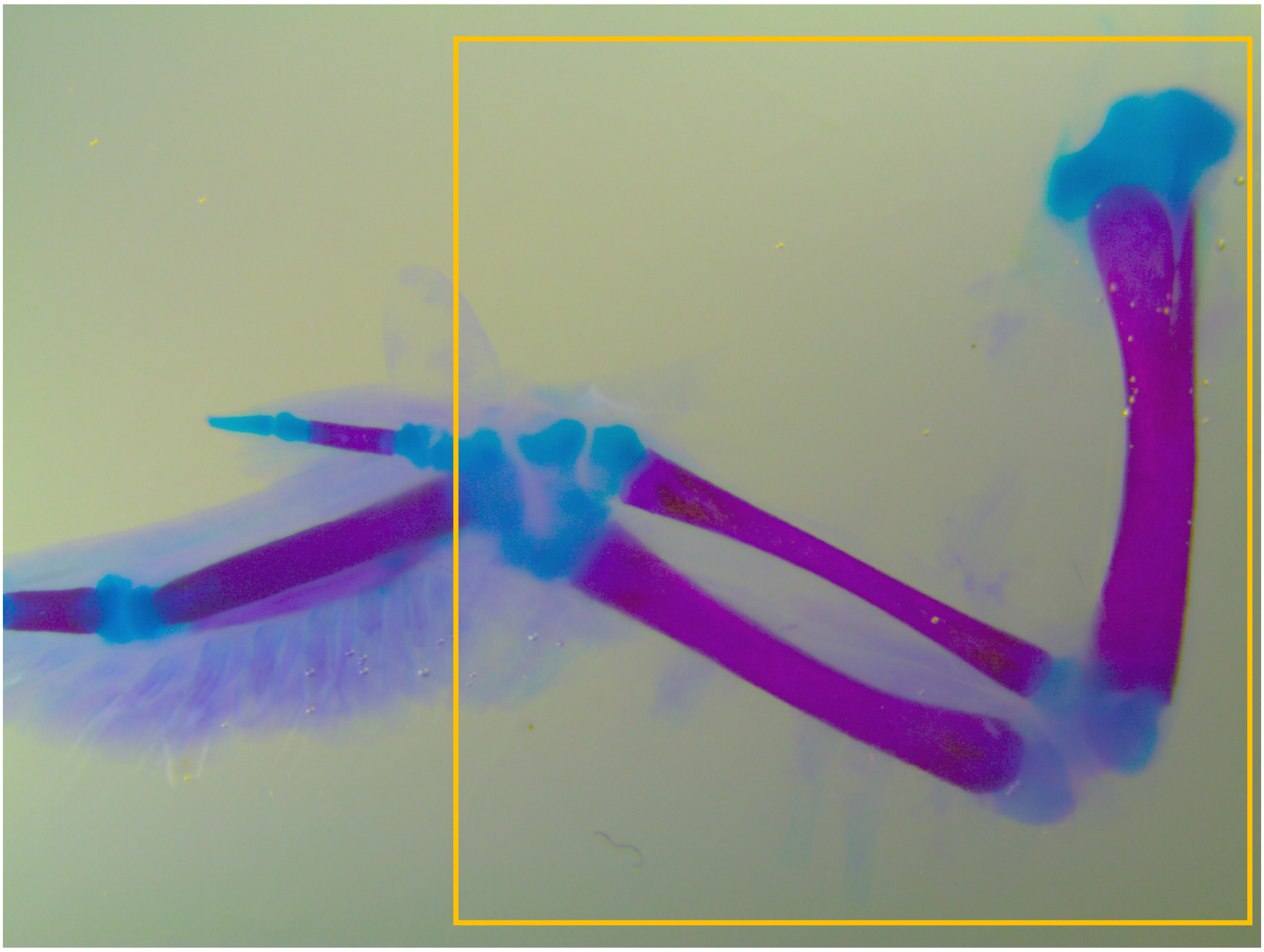

Figure 3D

Control pH2.5

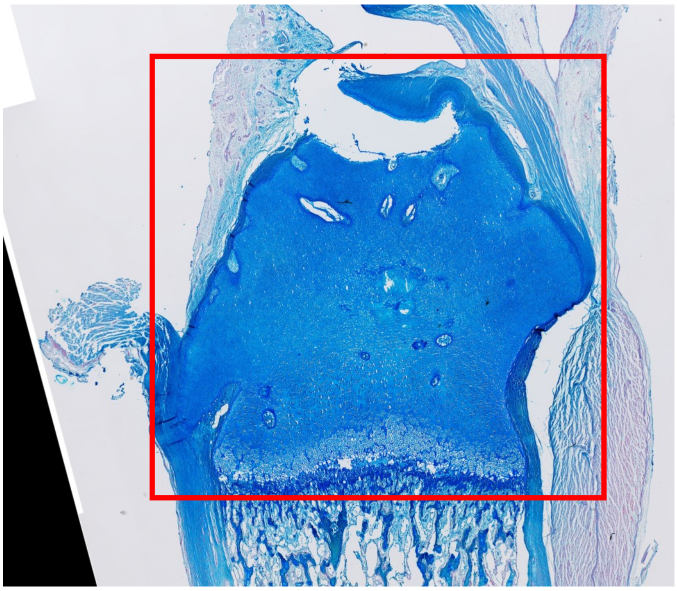

Control pH1.0

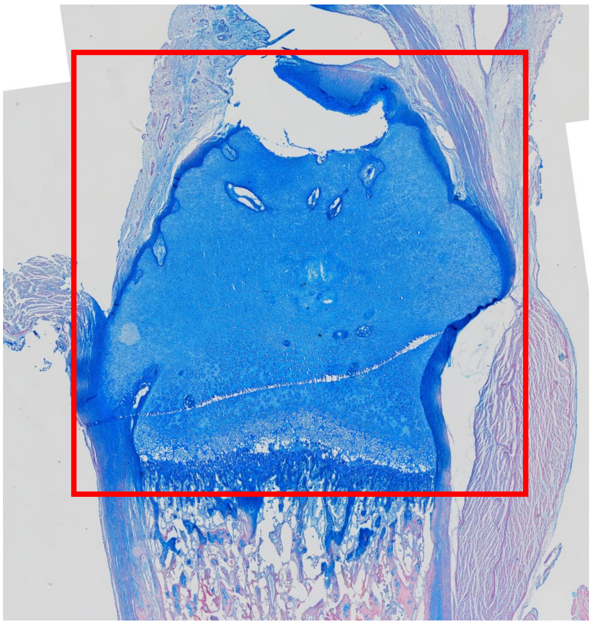

R05-365A pH2.5

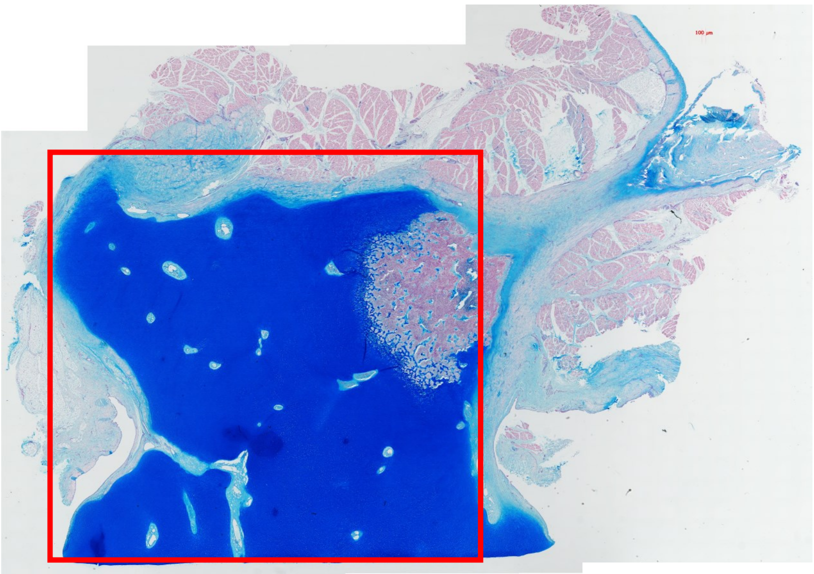

R05-365A pH1.0

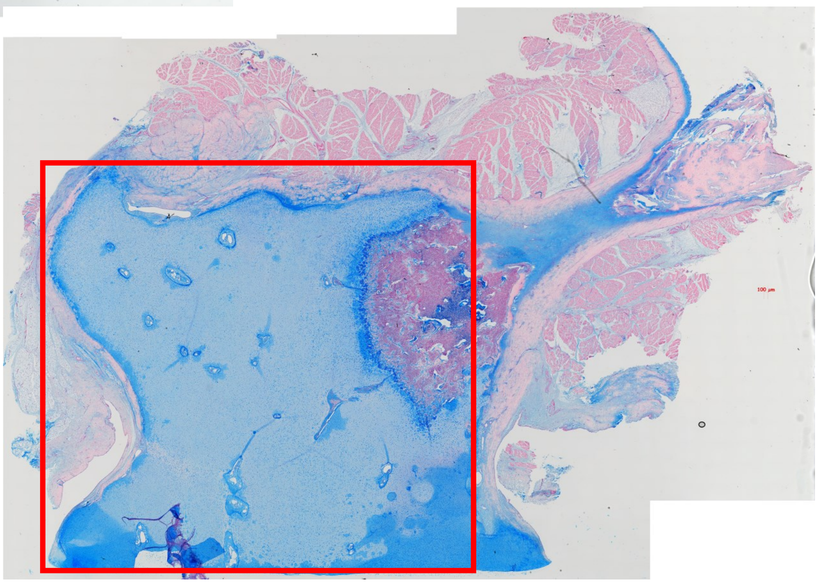

Figure 3E

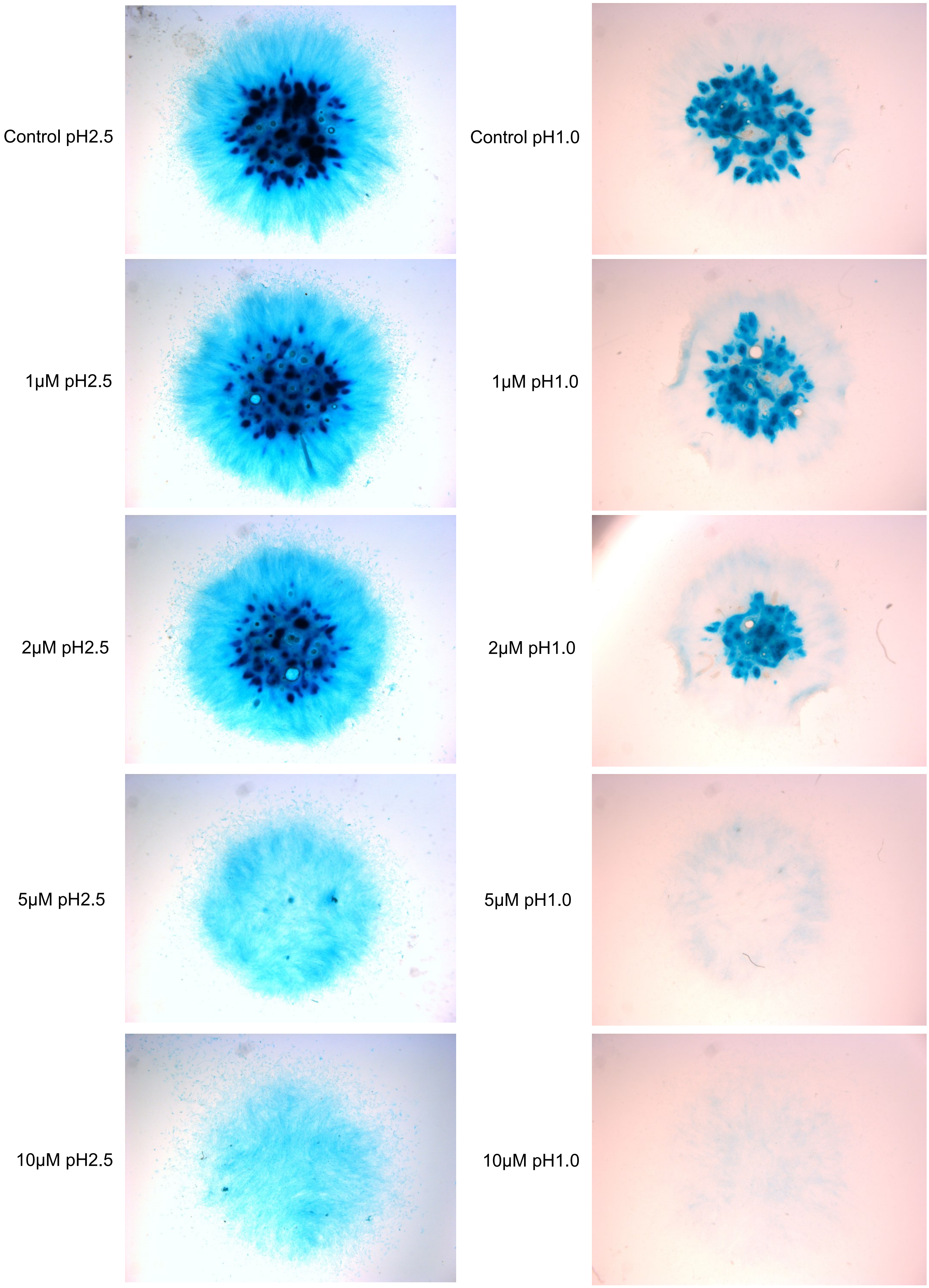
Control pH2.5
Control pH1.0
1μM pH2.5
1μM pH1.0
2μM pH2.5
2μM pH1.0
5μM pH2.5
5μM pH1.0
10μM pH2.5
10μM pH1.0

Figure 3F

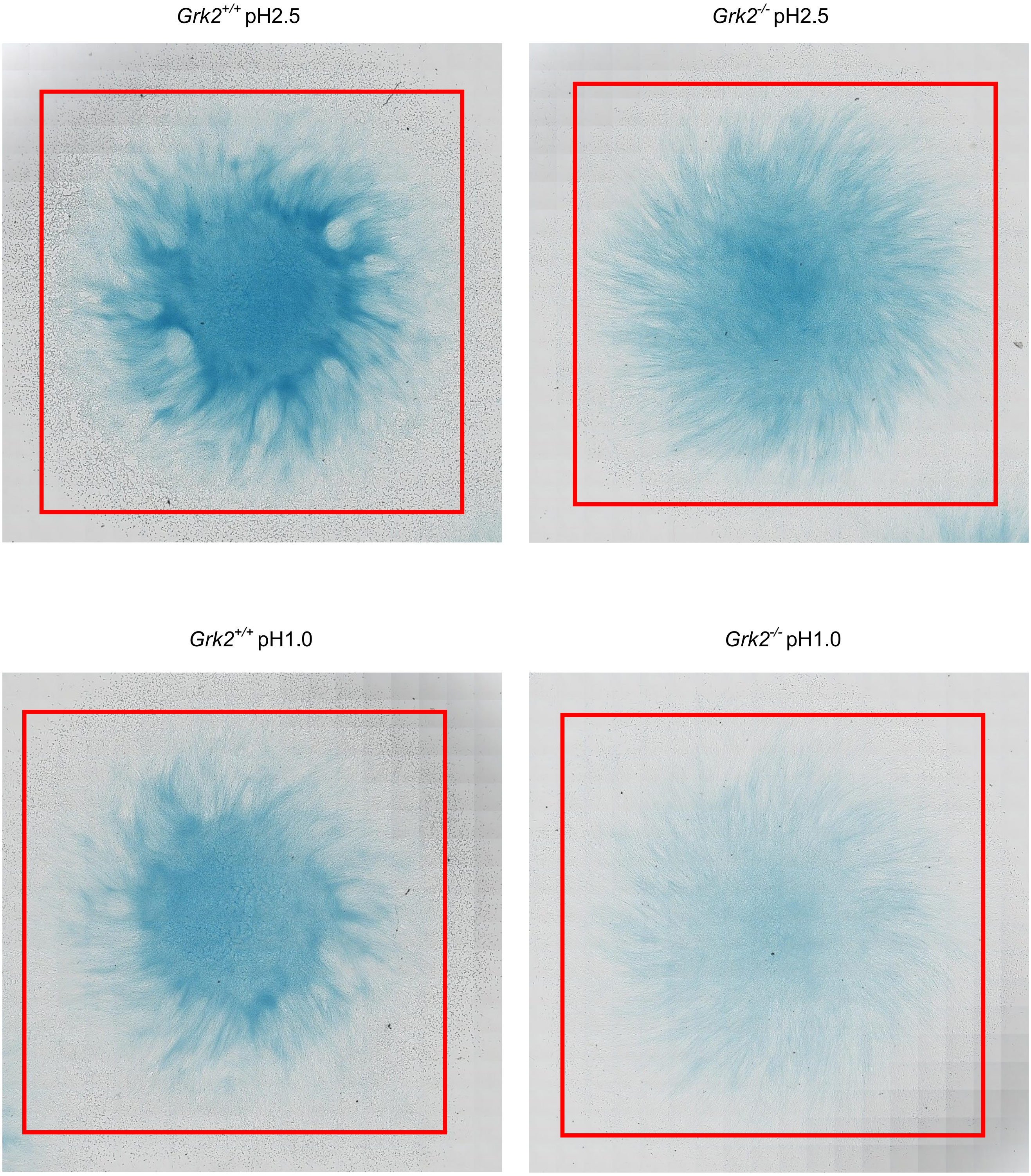

Supplement: Supplementary file 6 — Source Data for Figure 3 [file EMMM-12-e11739-s004.zip › EMM-2019-11739_SourceDataForFigure3/EMM-2019-11739_SourceDataForFigure3.pdf]
