# Supplementary material for: Mutations in GRK2 cause Jeune syndrome by impairing Hedgehog and canonical Wnt signaling
Source: EMBO Mol Med. 2020 Oct 14;12(11):e11739. doi: 10.15252/emmm.201911739 (PMC7645380; doi:10.15252/emmm.201911739)
Supplement: Supplementary file 8 — Source Data for Figure 5 [file EMMM-12-e11739-s006.zip › EMM-2019-11739_SourceDataForFigure5/EMM-2019-11739_SourceDataForFigure5.pdf]

Figure 5A

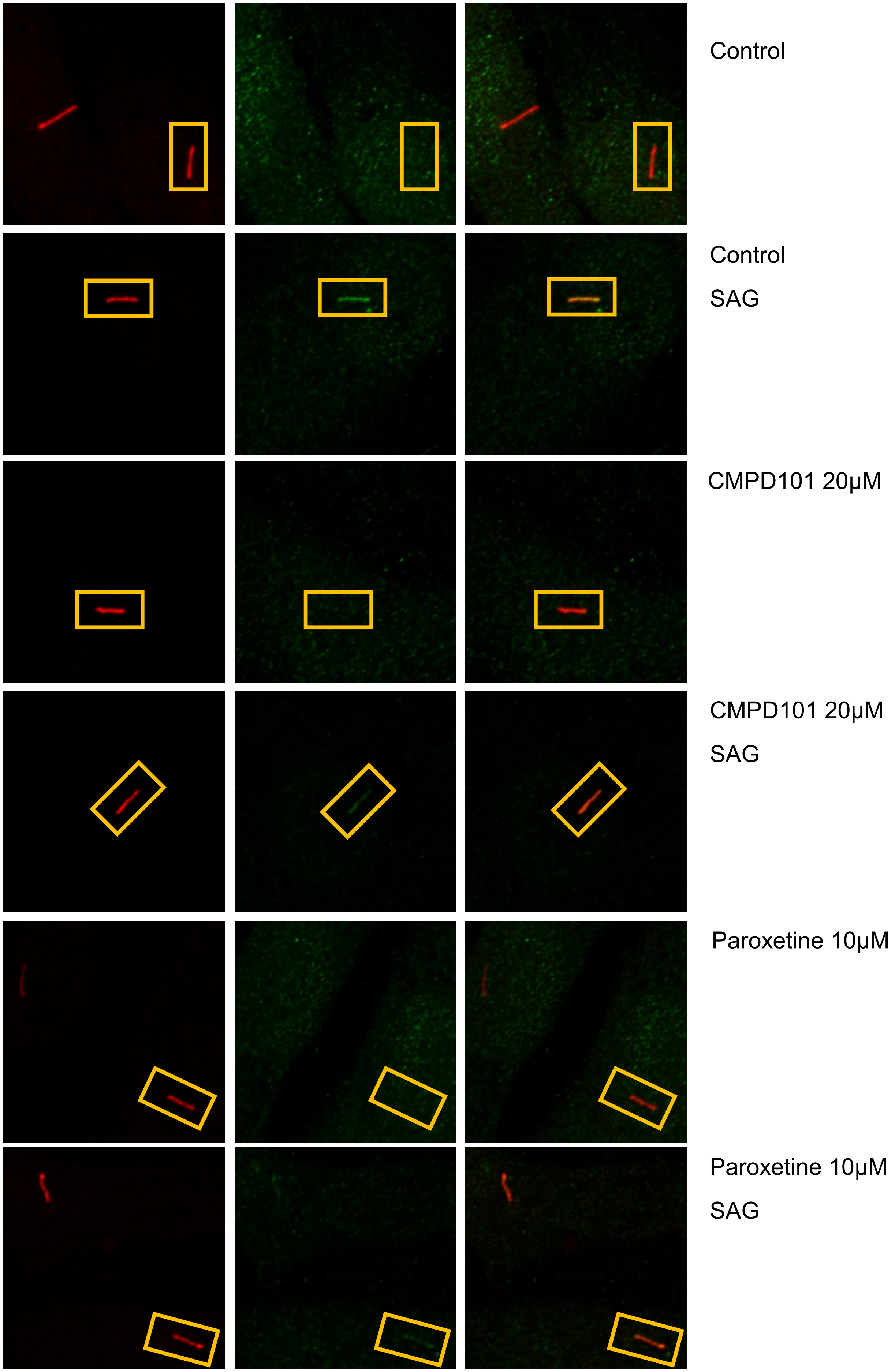
Control
Control
SAG
CMPD101 20μM
CMPD101 20μM
SAG
Paroxetine 10μM
Paroxetine 10μM
SAG

Figure 5C

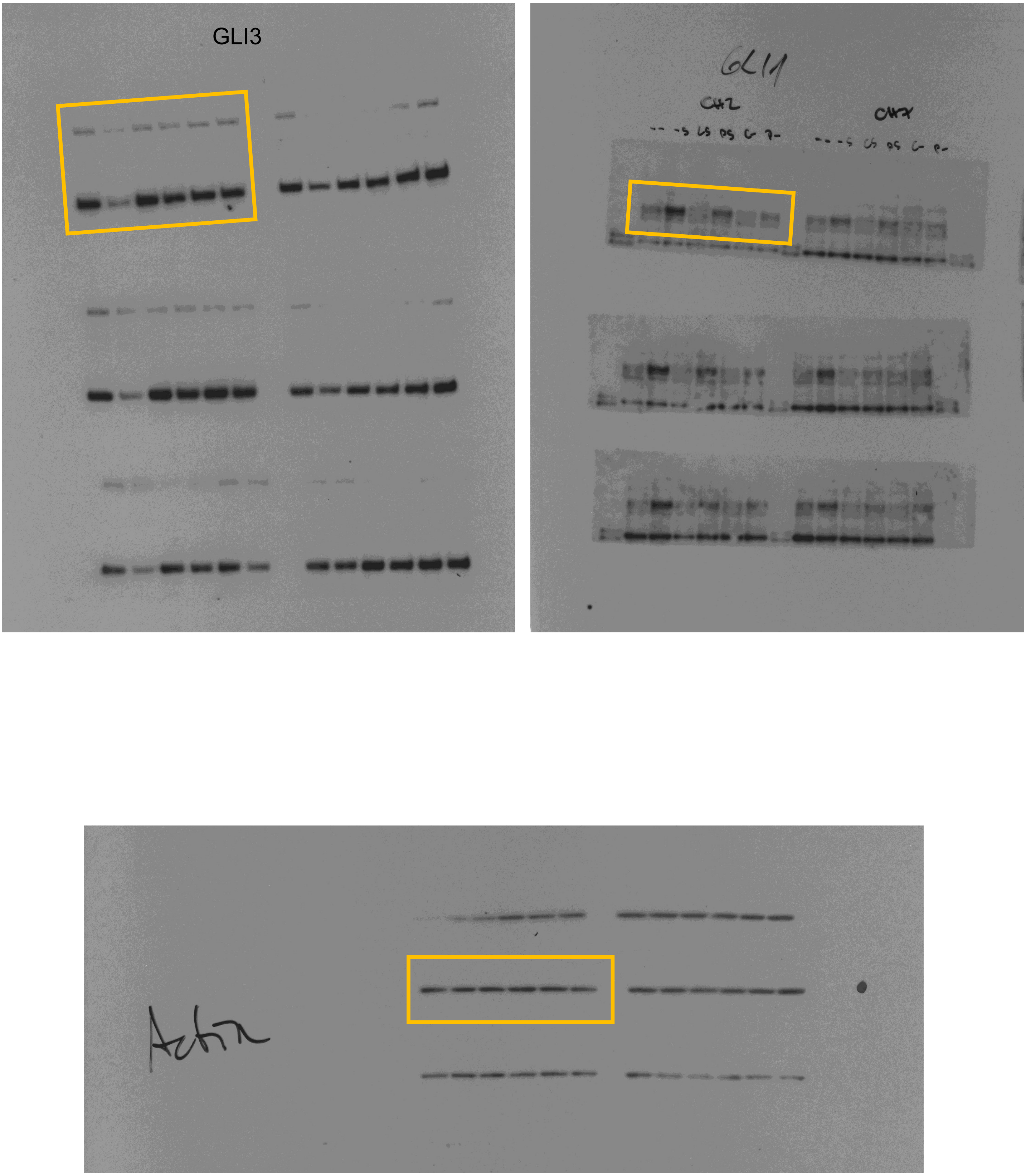

Figure 5E

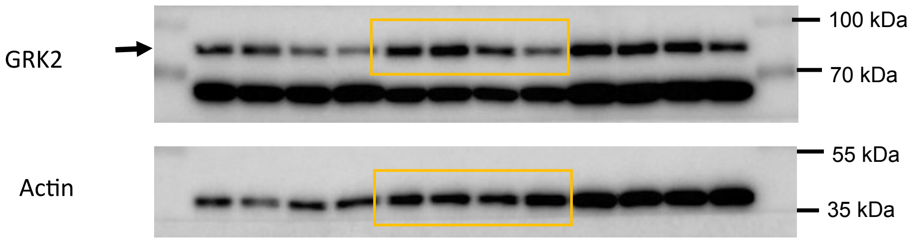

Figure 5F

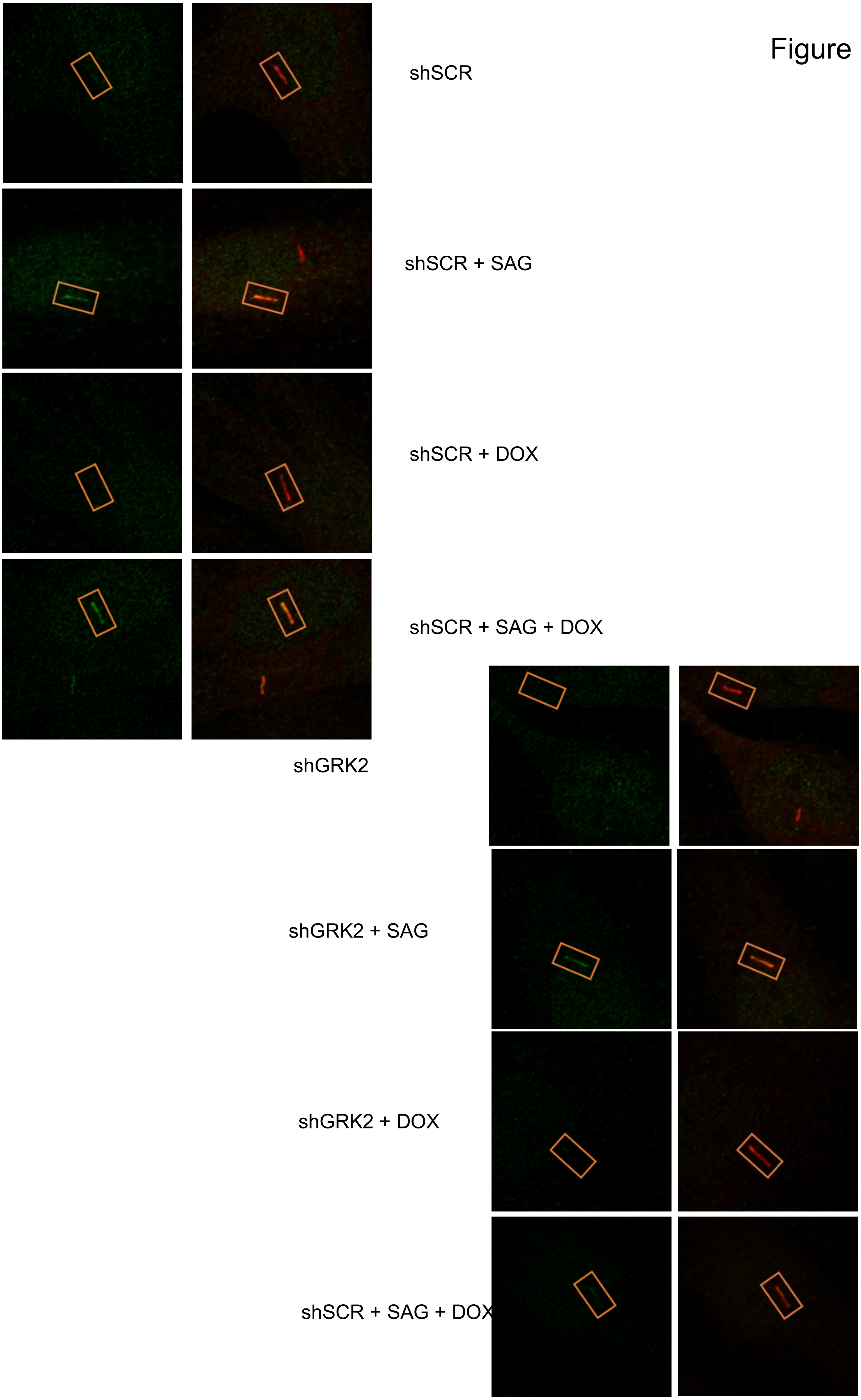
shSCR
shSCR + SAG
shSCR + DOX
shSCR + SAG + DOX
shGRK2
shGRK2 + SAG
shGRK2 + DOX
shSCR + SAG + DOX
