# Supplementary material for: Mutations in GRK2 cause Jeune syndrome by impairing Hedgehog and canonical Wnt signaling
Source: EMBO Mol Med. 2020 Oct 14;12(11):e11739. doi: 10.15252/emmm.201911739 (PMC7645380; doi:10.15252/emmm.201911739)

Figure EV2A

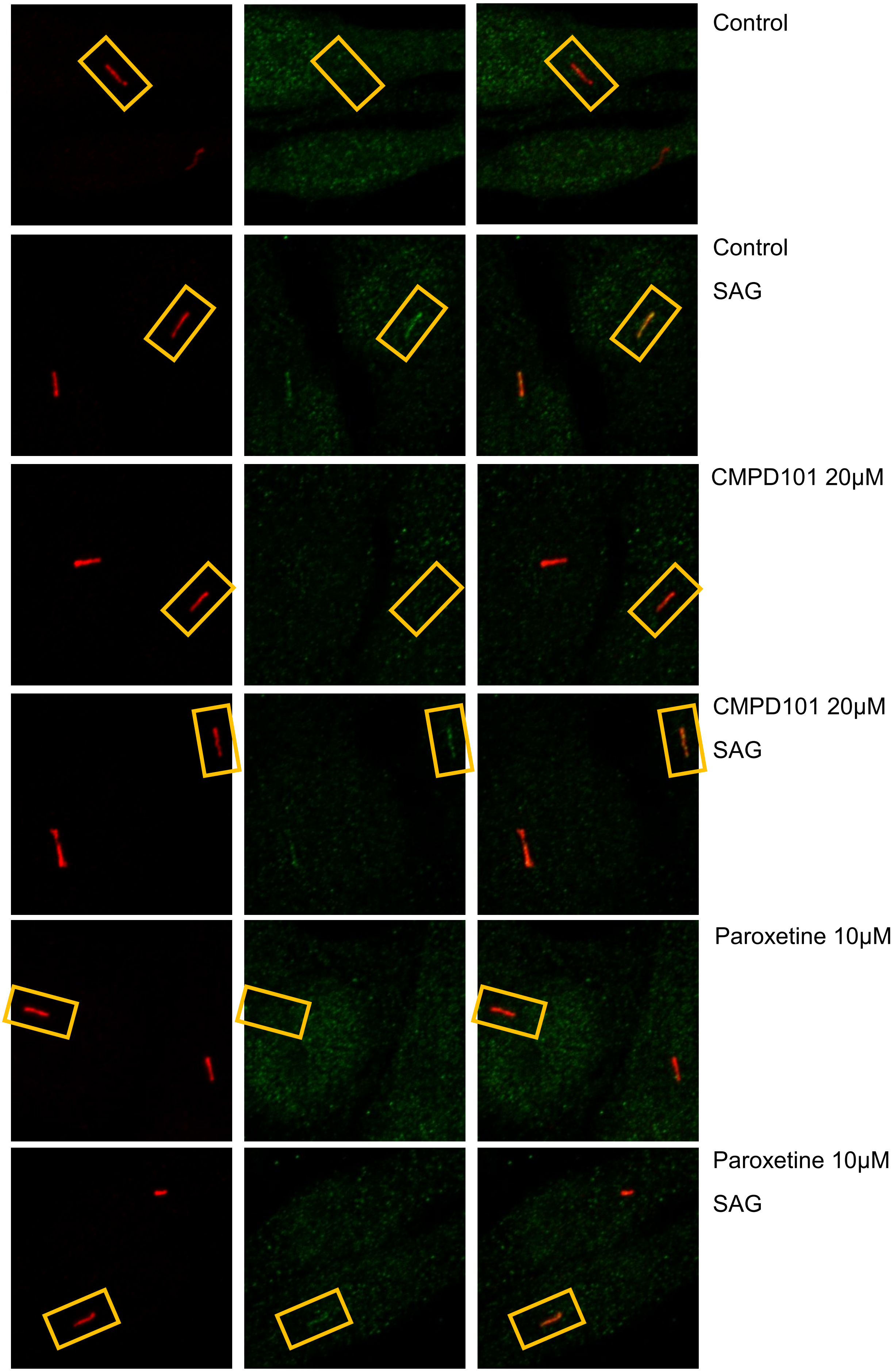
Control
Control
SAG
CMPD101 20μM
CMPD101 20μM
SAG
Paroxetine 10μM
Paroxetine 10μM
SAG

Figure EV2C

GLI3

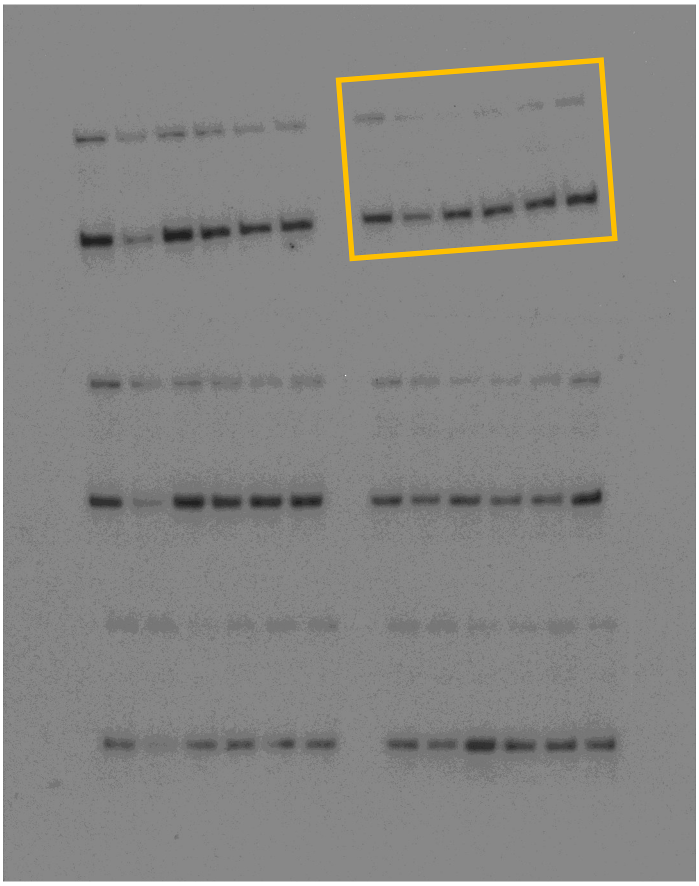

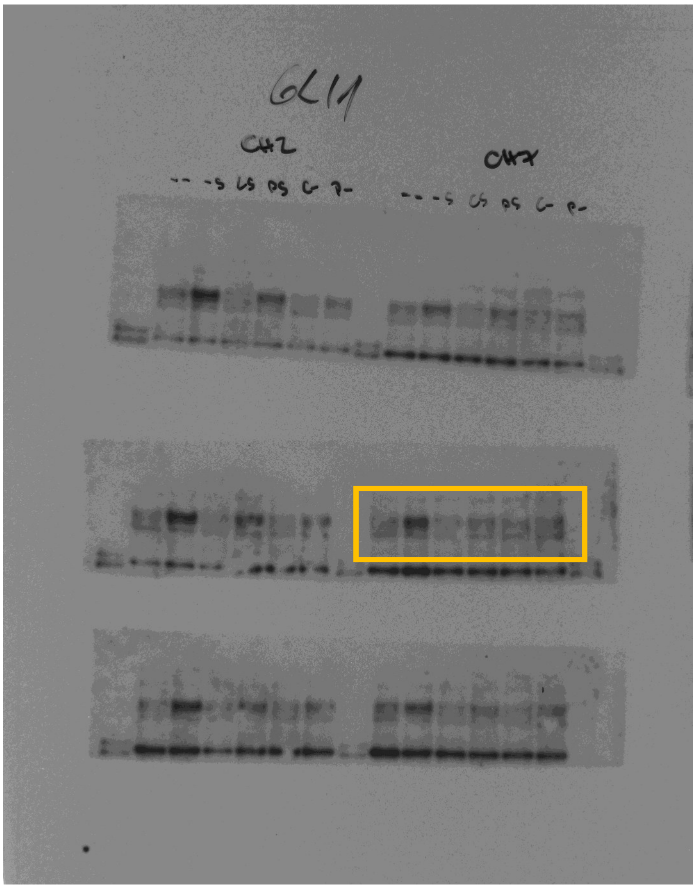

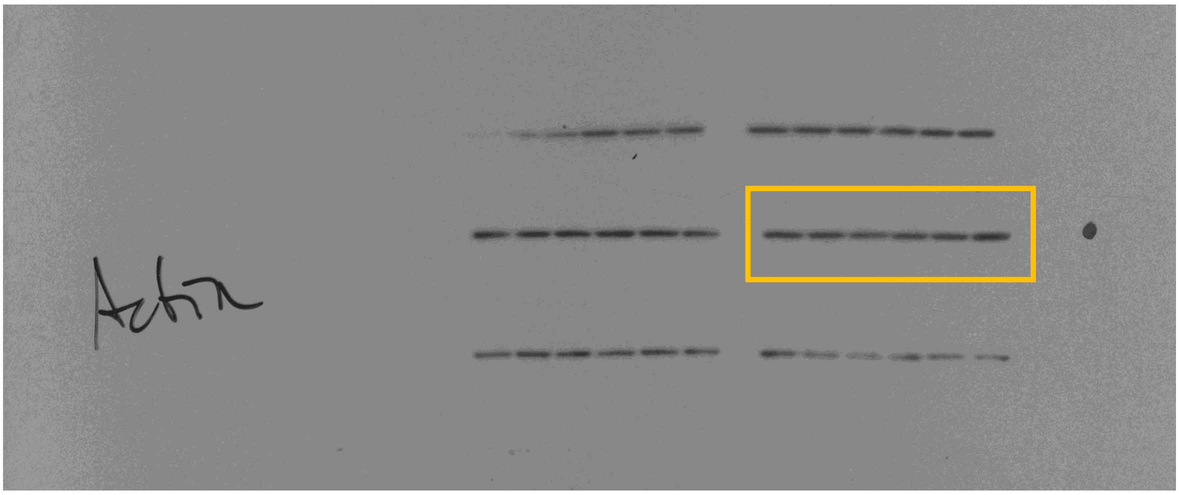

Supplement: Supplementary file 3 — Source Data for Expanded View [file EMMM-12-e11739-s009.zip › EMM-2019-11739_SourceDataForFigureEV2.zip!/EMM-2019-11739_SourceDataForFiguresEV2.pdf]
