# Supplementary material for: Mutations in GRK2 cause Jeune syndrome by impairing Hedgehog and canonical Wnt signaling
Source: EMBO Mol Med. 2020 Oct 14;12(11):e11739. doi: 10.15252/emmm.201911739 (PMC7645380; doi:10.15252/emmm.201911739)

Figure EV3A

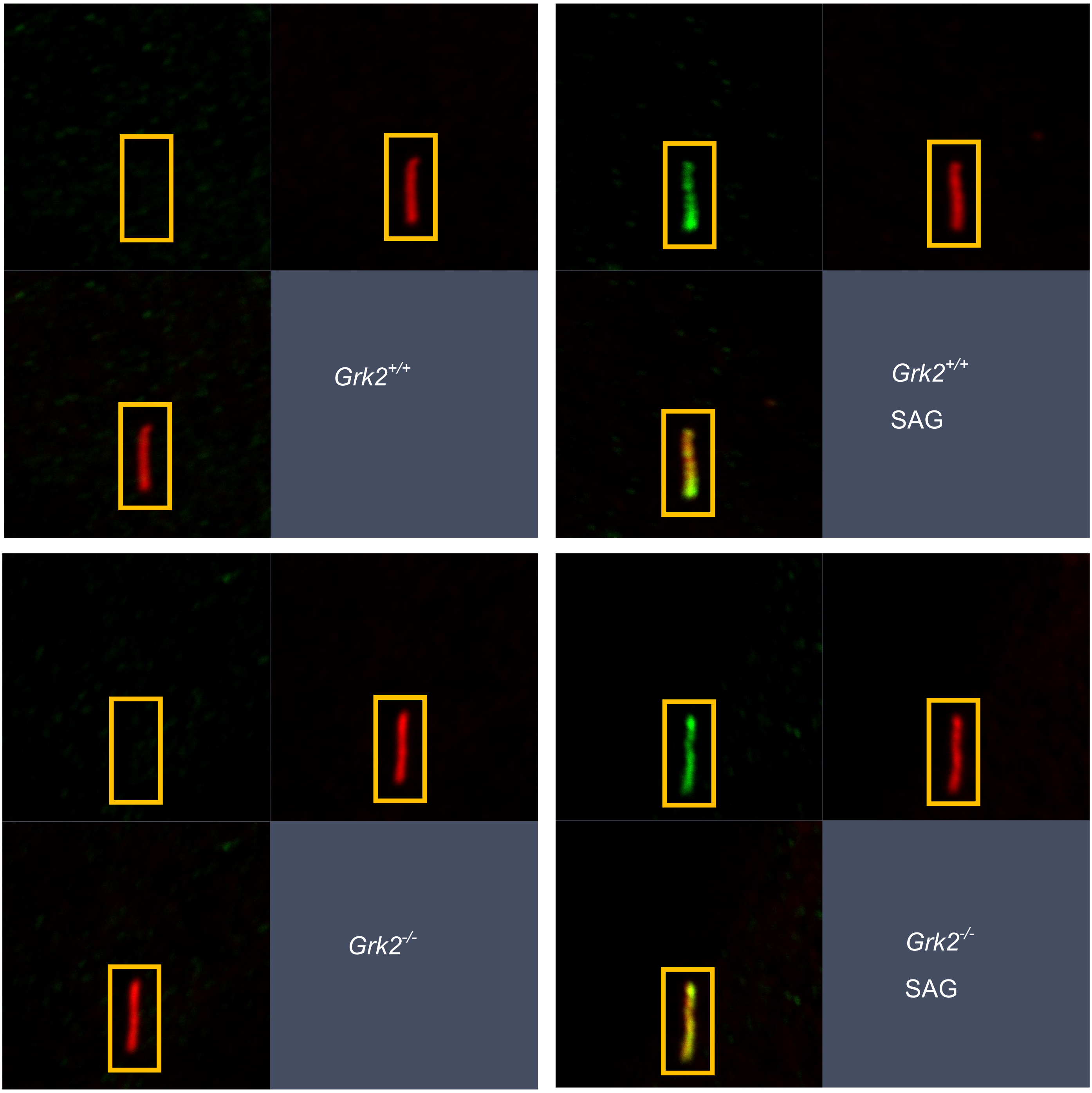

Figure EV3B

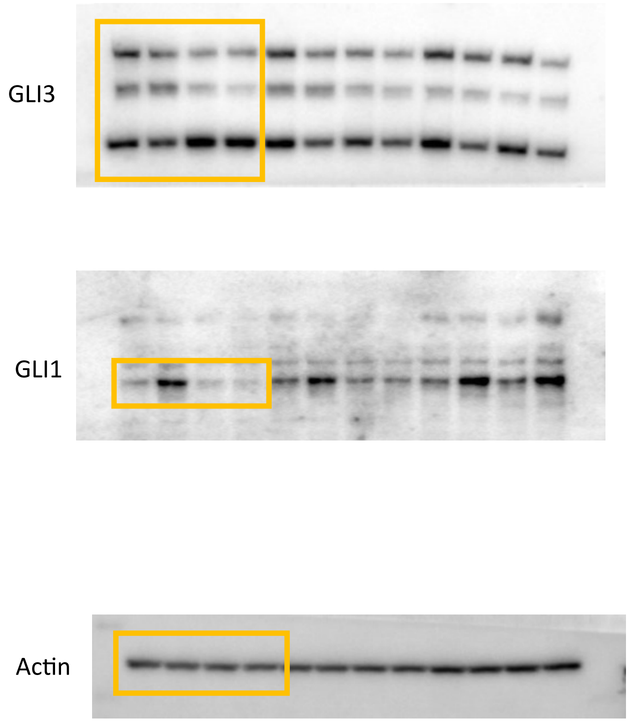

Figure EV3C

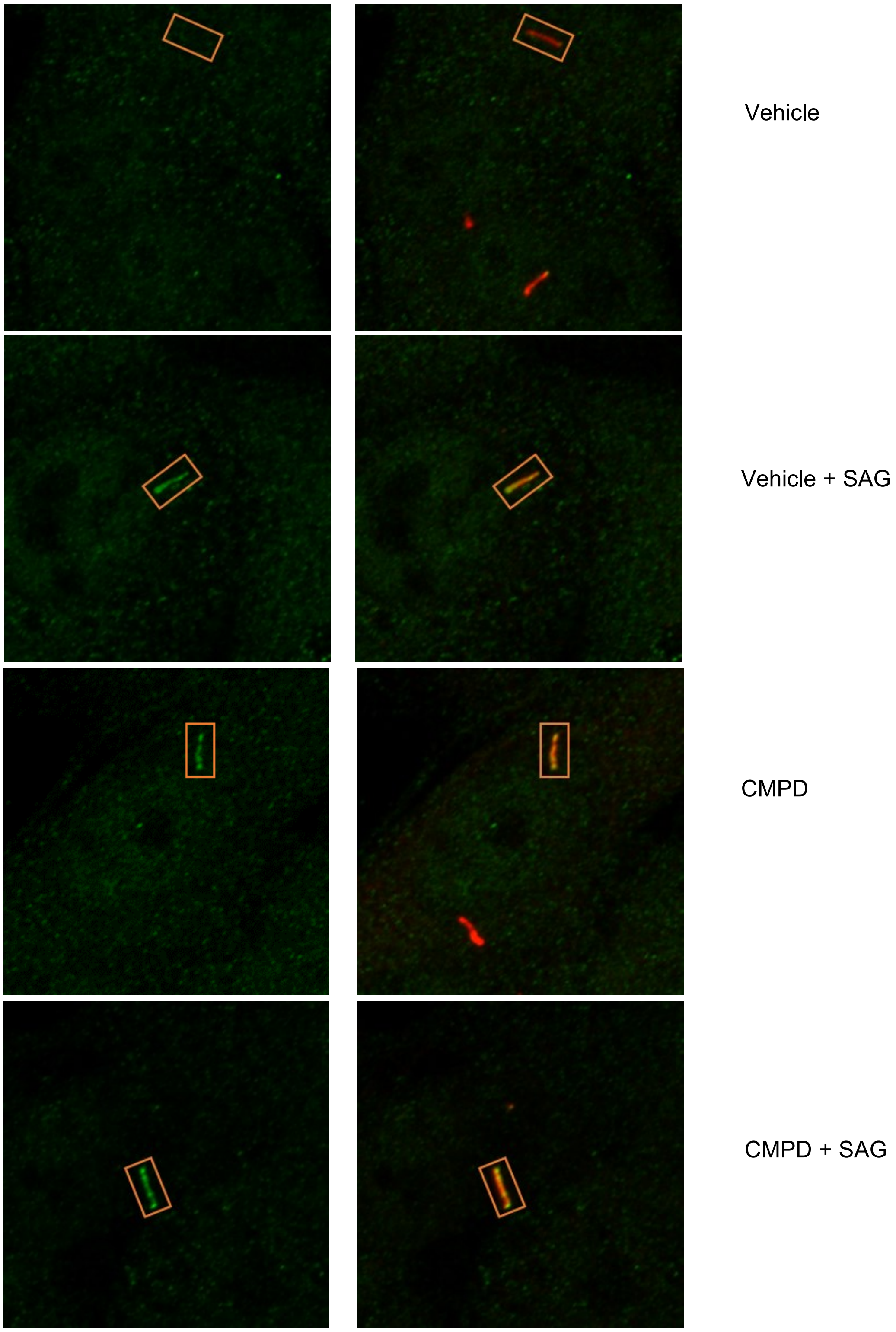

Figure EV3D

GLI3

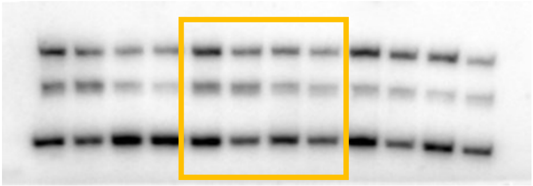

GLI1

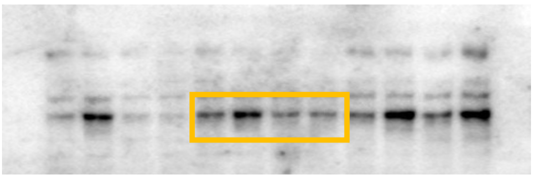

Actin

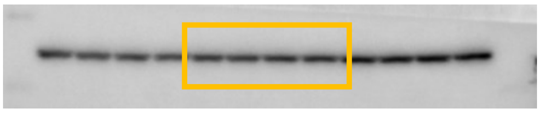

Figure EV3E

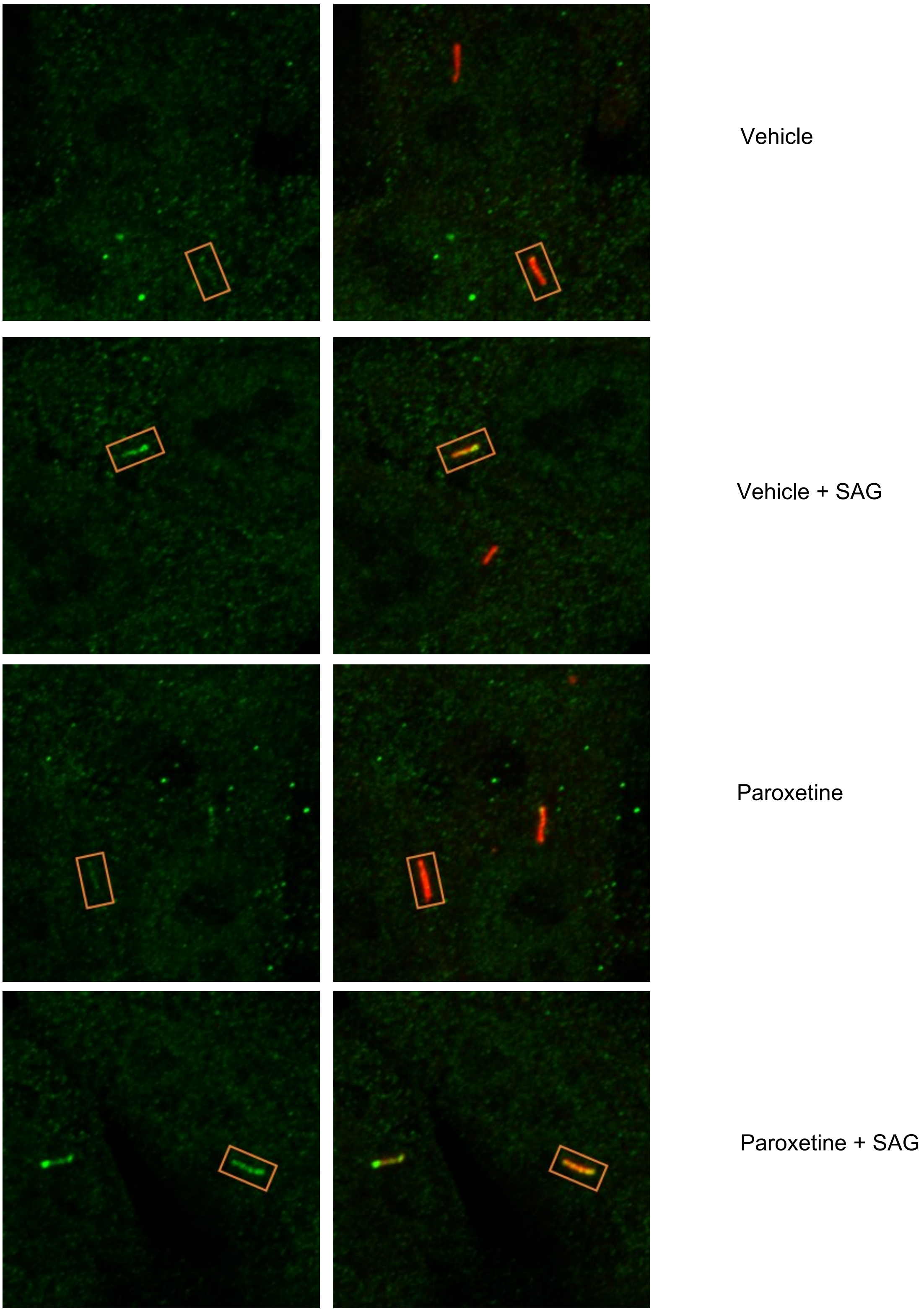

Figure EV3F

GLI3

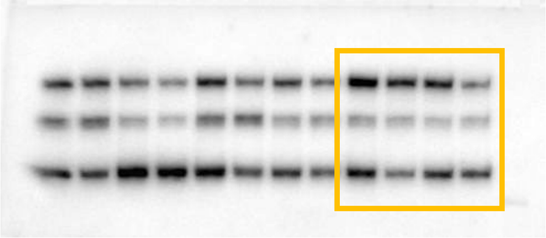

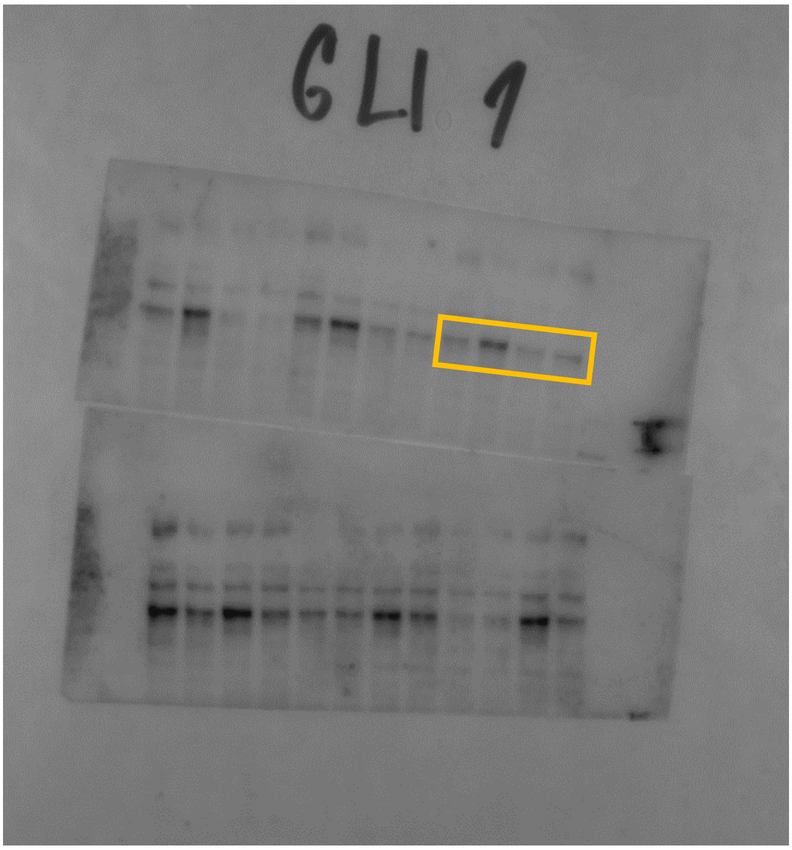

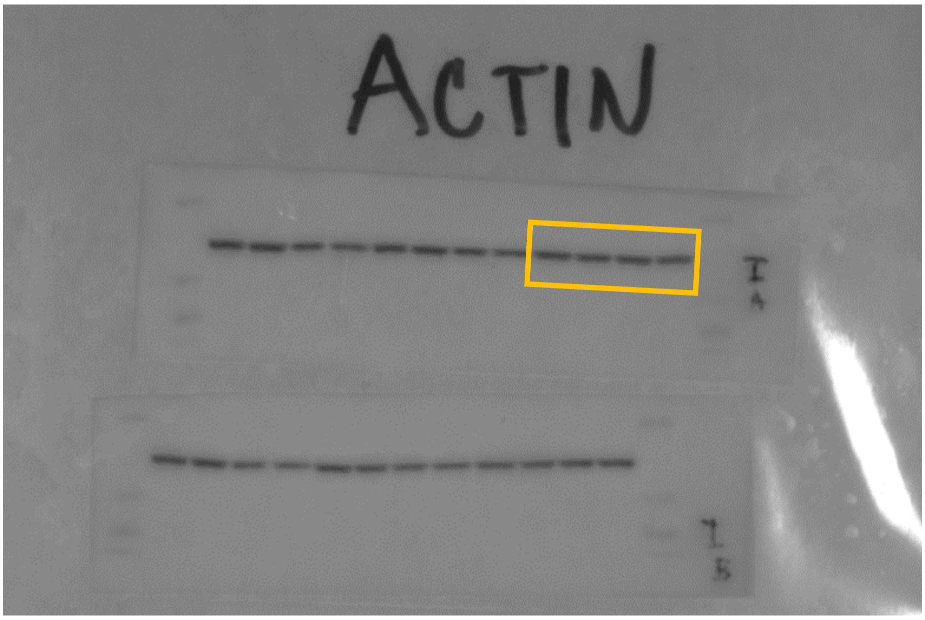

Figure EV3G

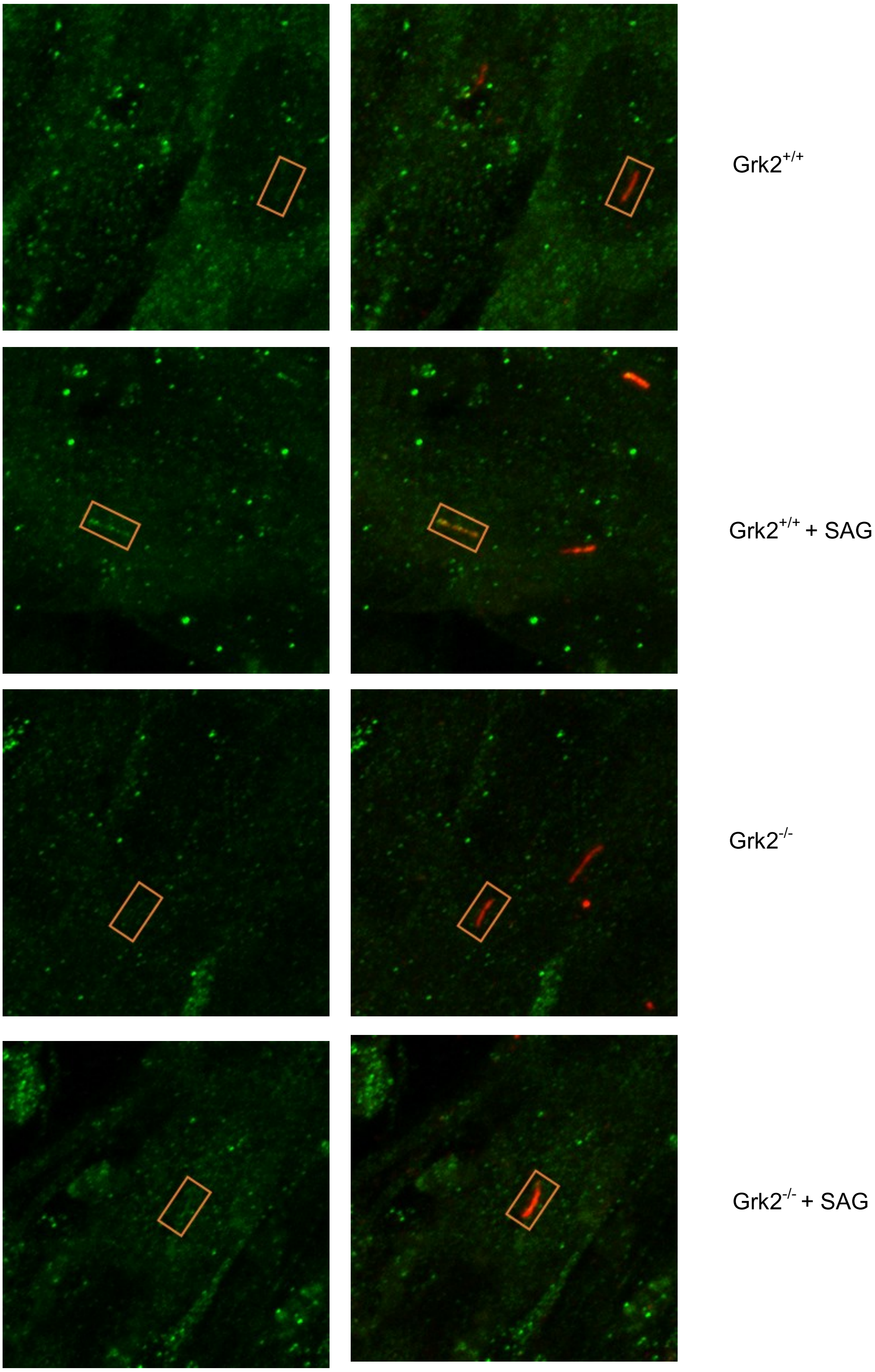

Figure EV3H

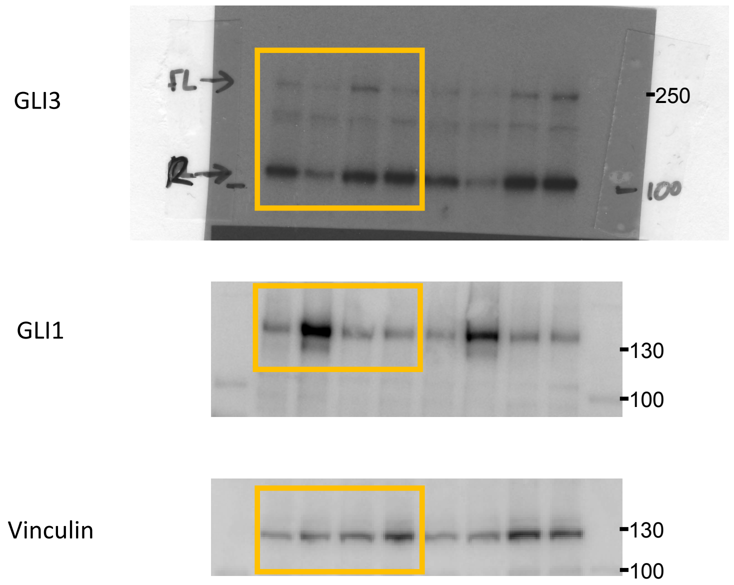

Figure EV3I

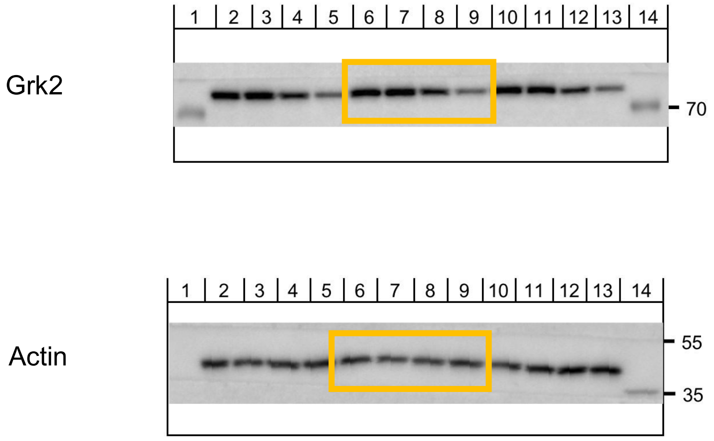
1
2
3
4
5
6
7
8
9
10
11
12
13
14
Grk2
70
1
2
3
4
5
6
7
8
9
10
11
12
13
14
Actin
55
35

Figure EV3J

shScr

shScr + SAG

shScr + Dox

shScr + SAG + Dox

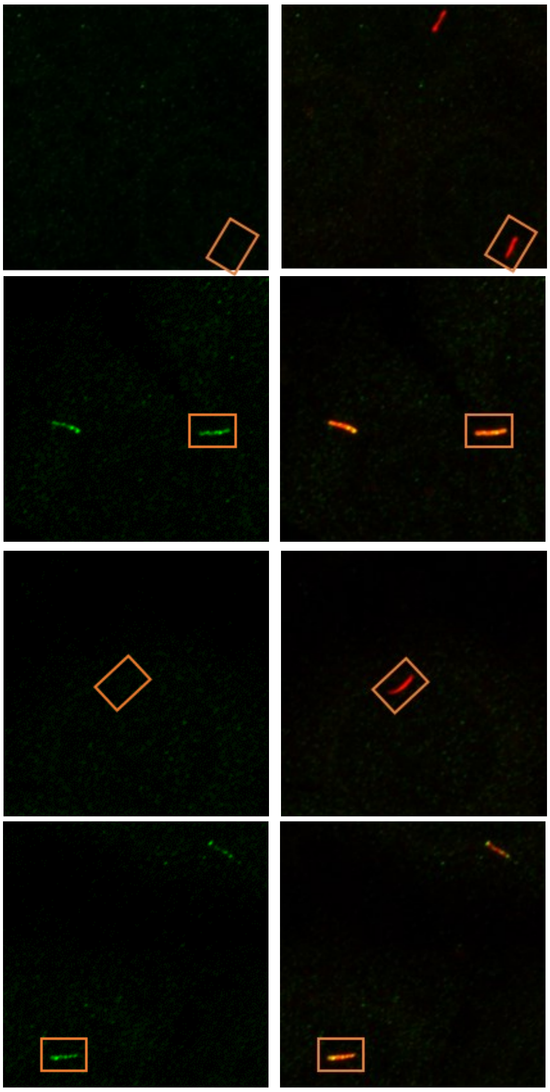

shGrk2

shGrk2 + SAG

shGrk2 + Dox

shGrk2 + SAG + Dox

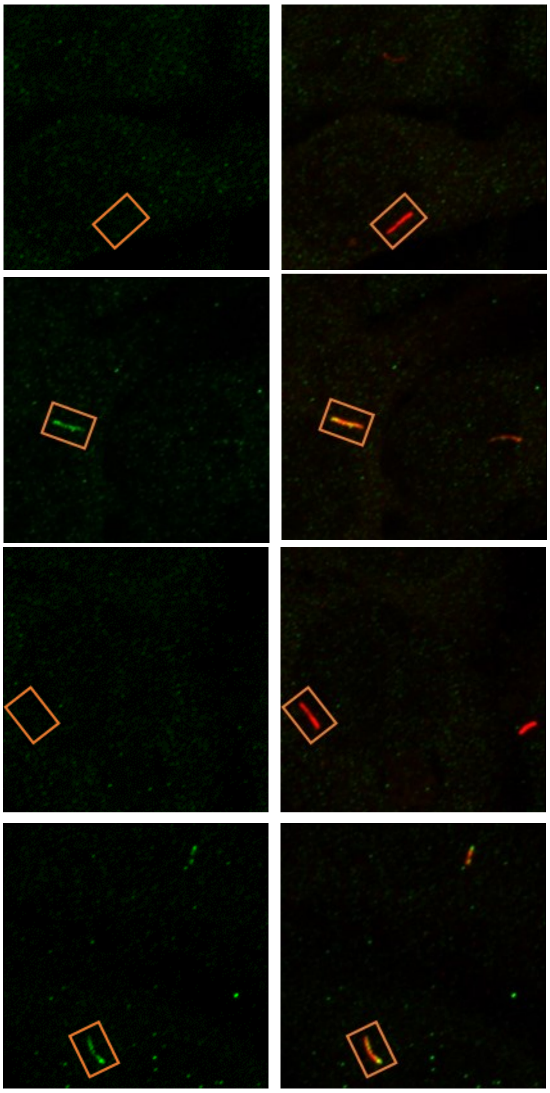

Figure EV3K

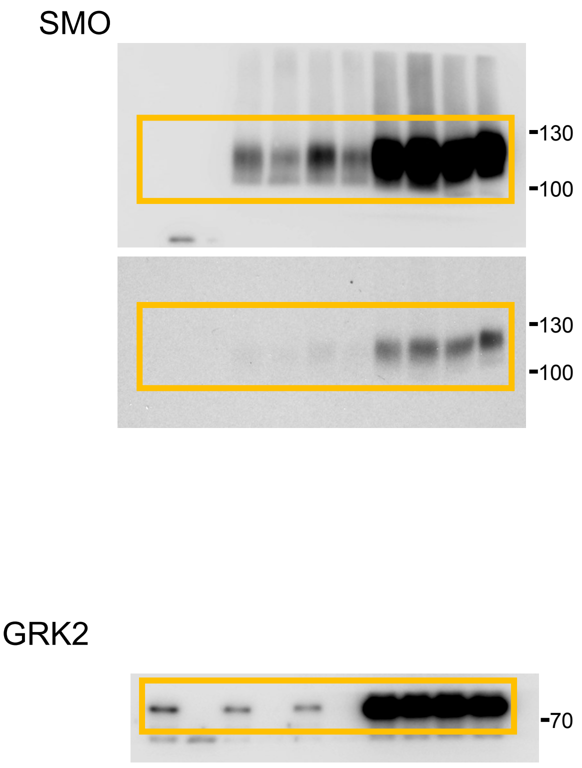

Supplement: Supplementary file 3 — Source Data for Expanded View [file EMMM-12-e11739-s009.zip › EMM-2019-11739_SourceDataForFigureEV3.zip!/EMM-2019-11739_SourceDataForFiguresEV3.pdf]
