# Supplementary material for: Mutations in GRK2 cause Jeune syndrome by impairing Hedgehog and canonical Wnt signaling
Source: EMBO Mol Med. 2020 Oct 14;12(11):e11739. doi: 10.15252/emmm.201911739 (PMC7645380; doi:10.15252/emmm.201911739)
Supplement: Supplementary file 3 — Source Data for Expanded View [file EMMM-12-e11739-s009.zip › EMM-2019-11739_SourceDataForFigureEV4.zip!/EMM-2019-11739_SourceDataForFiguresEV4.pdf]

Figure EV4B

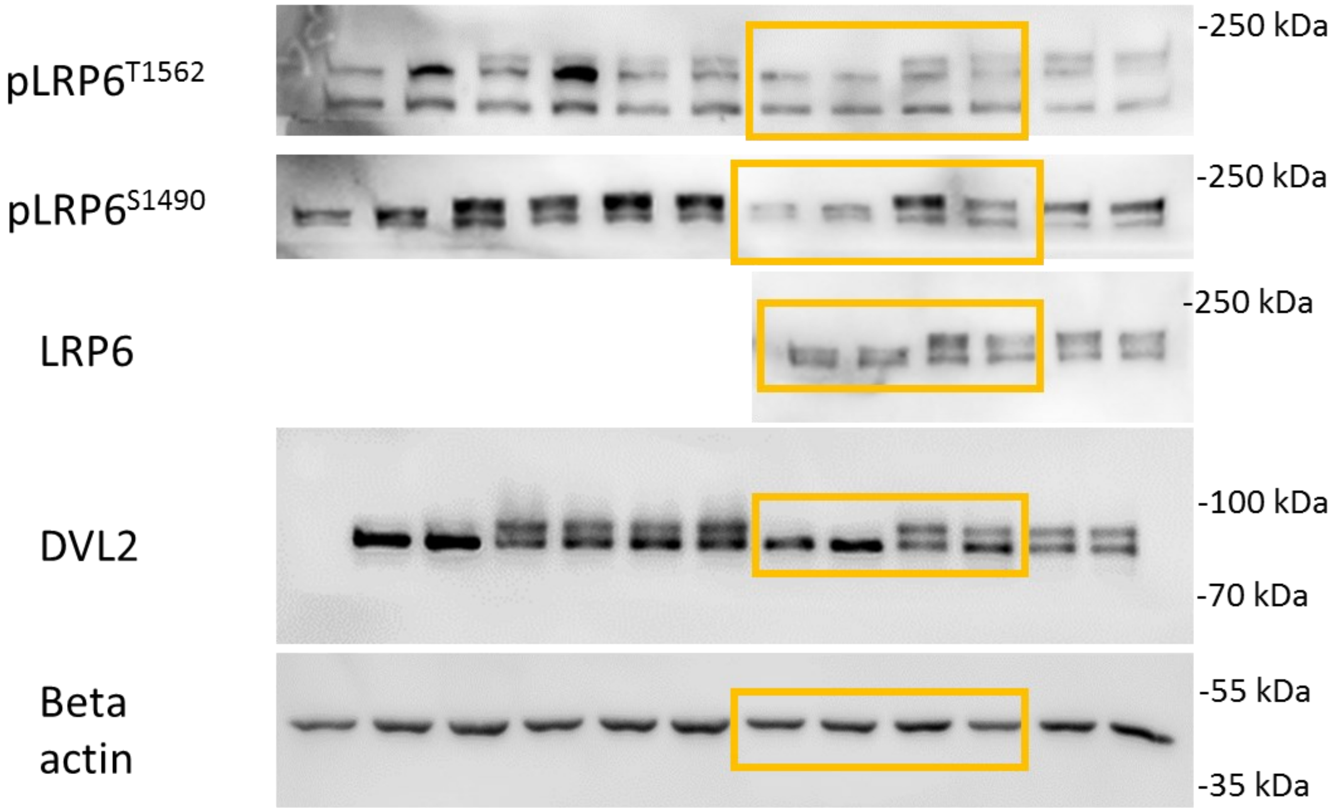

pLRP6T1562
pLRP6S1490
LRP6
DVL2
Beta actin
-250 kDa
-250 kDa
-250 kDa
-100 kDa
-70 kDa
-55 kDa
-35 kDa

Figure EV4C

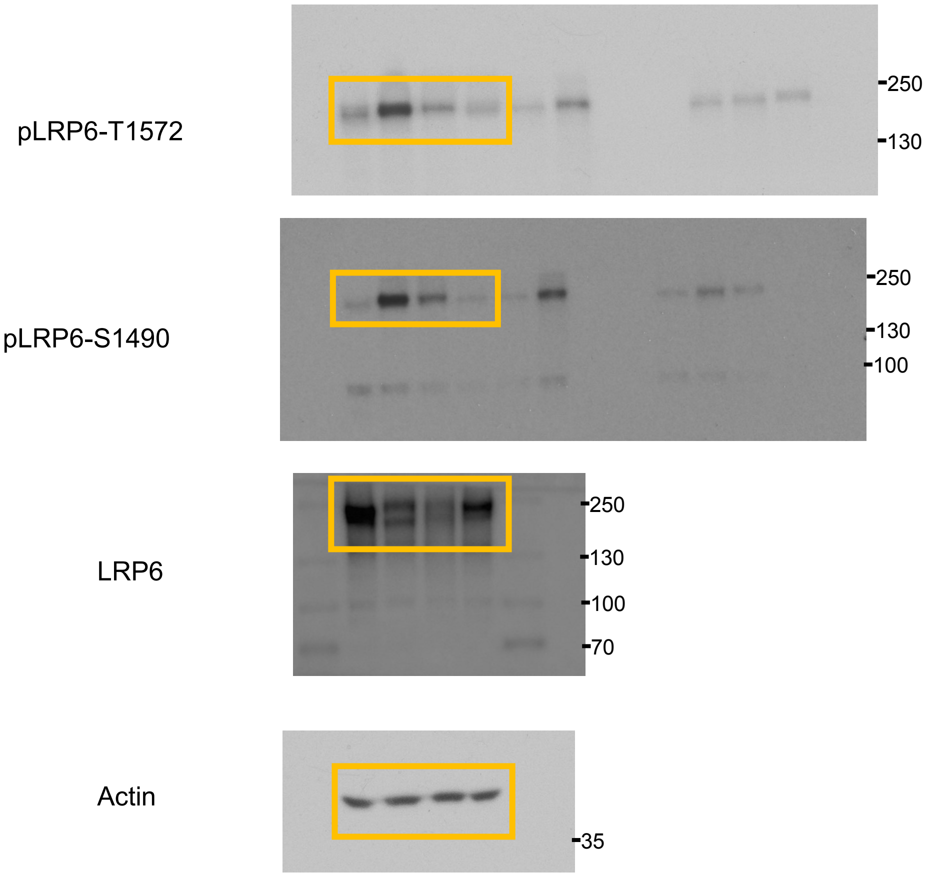

pLRP6-T1572
250
130
pLRP6-S1490
250
130
100
LRP6
250
130
100
70
Actin
35

Figure EV4D

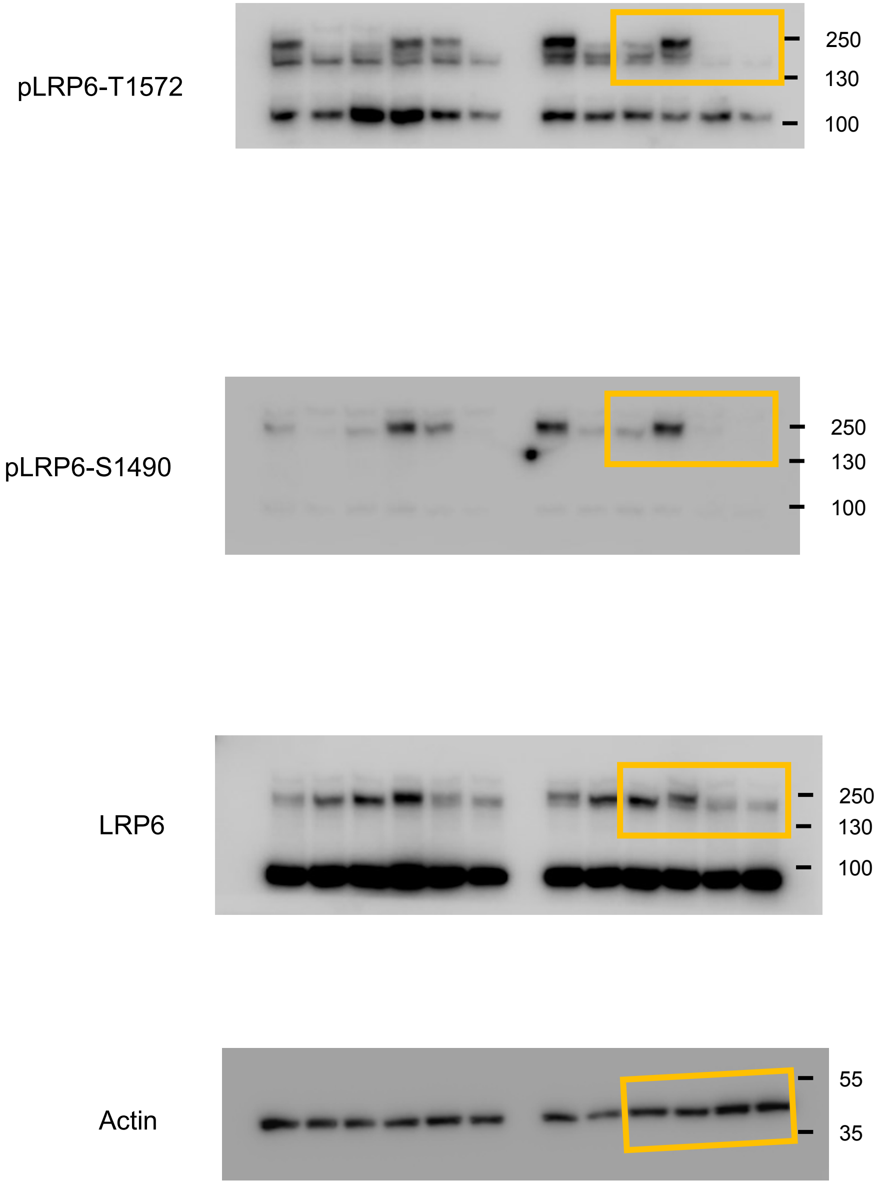
pLRP6-T1572
250
130
100
pLRP6-S1490
250
130
100
LRP6
250
130
100
Actin
55
35

Figure EV4E

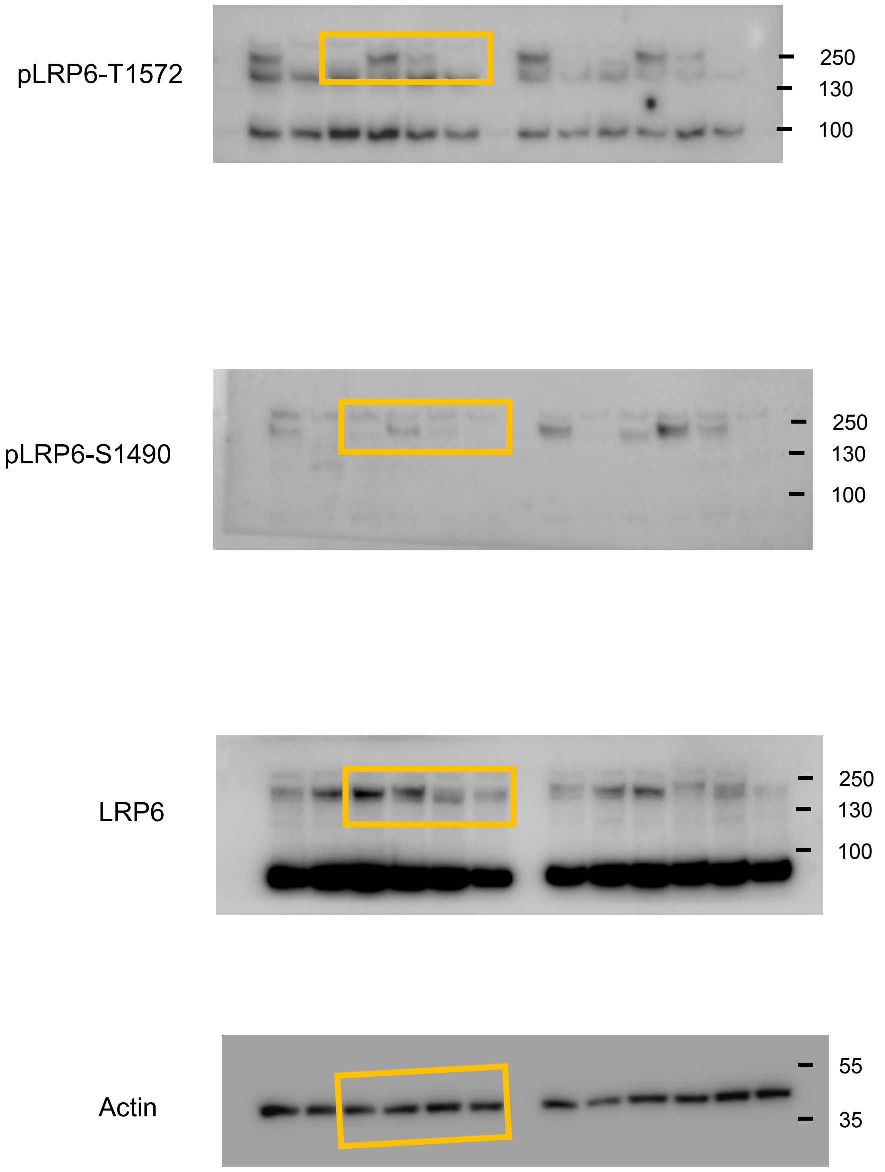
pLRP6-T1572
250
130
100
pLRP6-S1490
250
130
100
LRP6
250
130
100
Actin
55
35
